# Response of *Saccharomyces cerevisiae* to the Stimulation of Lipopolysaccharide

**DOI:** 10.1371/journal.pone.0104428

**Published:** 2014-08-08

**Authors:** Lulu Shen, Ye Li, Linghuo Jiang, Xiaoyuan Wang

**Affiliations:** 1 State Key Laboratory of Food Science and Technology, Jiangnan University, Wuxi, China; 2 School of Biotechnology, Jiangnan University, Wuxi, China; 3 Synergetic Innovation Center of Food Safety and Nutrition, Jiangnan University, Wuxi, China; CNR, Italy

## Abstract

Lipopolysaccharide, known as endotoxin, can stimulate potent host immune responses through the complex of Toll-like-receptor 4 and myeloid differentiation protein 2; but its influence on *Saccharomyces cerevisiae*, a model organism for studying eukaryotes, is not clear. In this study, we found that lipopolysaccharide-treated *S. cerevisiae* cells could be stained by methylene blue, but did not die. Transcriptional profiling of the lipopolysaccharide-treated *S. cerevisiae* cells showed that 5745 genes were modulated: 2491 genes up-regulated and 3254 genes down-regulated. Significantly regulated genes (460 up-regulated genes and 135 down-regulated genes) in lipopolysaccharide-treated *S. cerevisiae* cells were analyzed on Gene Ontology, and used to establish physical protein-protein interaction network and protein phosphorylation network. Based on these analyses, most of the regulated genes in lipopolysaccharide-treated *S. cerevisiae* cells were related to cell wall, membrane, peroxisome and mitochondrion. Further experiments demonstrated that lipopolysaccharide stimulation caused the exposure of phosphatidylserine and the increase of mitochondrial membrane potential in *S. cerevisiae* cells, but levels of intracellular reactive oxygen species and metacaspase activation were not increased. This study demonstrated that lipopolysaccharide stimulation causes significant changes in *S. cerevisiae* cells, and the results would contribute to understand the response of eukaryotic cells to lipopolysaccharide stimulation.

## Introduction

*Saccharomyces cerevisiae* is a model organism commonly used to study many obscure aspects of important human pathologies that are harder to elucidate using other more complex eukaryotic models [1], [2], [3]. Different stresses cause different responses in *S. cerevisiae* and usually result in cell death [3], [4]. For example, heat shock, oxidative stress and reagents such as formic acid and aspirin commit apoptosis in *S. cerevisiae* cells [5]–[8], while leucine starvation and the pharmacological reagent rapamycin cause autophagy [9]–[11].

Lipopolysaccharide (LPS), known as endotoxin, is an essential component of the outer membrane in most Gram-negative bacteria, and it consists of a hydrophobic moiety termed as lipid A and a large polysaccharide subdivided into the nonrepeating “core” oligosaccharide and the O-antigen repeats [12], [13]. LPS has profound effect on the mammalian immune system and great significance in the pathophysiology of many disease processes [14]–[16]. As a pathogen-associated antigen, LPS can be recognized by different immune cells through a protein complex consisting of Toll-like receptor 4 and myeloid differentiation protein 2, trigger the production and release of pro-inflammatory cytokines, and activate the immune cells in inflammatory systems [14].

Considering the similarity between yeast and mammalian cells, the response of yeast to LPS stimulation might provide important information on the signal transducing mechanisms underlying inflammatory responses in mammalian cells, and assist in the search and screening of new potential anti-inflammatory drugs. However, there are only a couple of papers published on the response of yeast to LPS. One study concluded that *S. cerevisiae* is able to respond to the presence of LPS and that Hog1 was phosphorylated and a few genes such as *GPD1* were up-regulated in cells exposed to LPS [17]. To understand the detailed mechanism on the response of *S. cerevisiae* to LPS, we systematically investigated LPS-treated *S. cerevisiae* BY4742 cells using transcriptome analysis and different staining methods.

## Material and Methods

### Methylene blue staining and cell viability determination

*S. cerevisiae* BY4742 was grown in YPD media (2% tryptone, 1% yeast extract, 2% glucose) or SD media (0.67% yeast nitrogen base, 2% glucose) supplemented with appropriate amino acids at 30°C. *S. cerevisiae* cells were harvested at the pre-log phase, and resuspended in phosphate-buffered saline (PBS, pH7.4). LPS from *Escherichia coli* O111:B4 (Sigma-Aldrich) was dissolved in PBS at 2 mg/mL and mixed with the cell suspensions. The optical density at 600 nm (OD_600_) of the mixture was adjusted to 2.0. The mixture was incubated at 30°C. When SD media were used to replace PBS in some experiments, 0.5 mg/mL LPS was used, and OD_600_ of the mixture was adjusted to 1.0.

Methylene blue (Sinopharm Chemical Reagent) was used to stain *S. cerevisiae* cells after treated with different concentrations of LPS. Three different methylene blue solutions with different pH were prepared. The first solution contains 0.01% methylene blue and 2% sodium citrate [18]; the second solution was prepared by dissolving 0.3 g methylene blue in 30 mL 95% EtOH mixed with 70 mL 0.01% KOH [19]; the third solution contains 0.02% methylene blue, 2.72% KH_2_PO_4_, and 0.072‰ Na_2_HPO_4_ [20]. *S. cerevisiae* cell suspensions were mixed with equal volume of the methylene blue solution for 3 min [19] or 10 min [18], [20] and the stained cells were observed under light microscopy.

To quantify the number of viable yeast cells in the mixture of *S. cerevisiae* cells with or without LPS, serial dilutions of the mixture were dropped on YPD agar plate, and colonies were counted after 3-day's incubation at 30°C.

### RNA preparation and the whole genome transcriptional analysis

The whole genome analysis of gene expression was established according to the published method [17]. A single colony of *S. cerevisiae* cells was inoculated in SD media supplemented with appropriate amino acids. When OD_600_ reached 1.0, cells were divided and incubated with or without 0.5 mg/mL LPS in SD media. After incubation at 30°C for 90 min or 180 min with gentle shaking, cells were harvested, and the total RNAs were extracted immediately [21]. The RNA extracts were treated with DNase I to remove DNA and isolate mRNA. Then cDNA was synthesized using the mRNA as templates. During the quantity control steps, Agilent 2100 Bioanaylzer (Agilent Technologies) and ABI StepOnePlus Real-Time PCR System (Life Technologies) were used in quantification and qualification of the sample library. The library was sequenced using Illumina HiSeq 2000 (BGI-Shenzhen, China) [22].

The physical protein-protein interactions (PPPI) networks in LPS-treated *S. cerevisiae* were constructed using Cytoscape software (http://www.cytoscape.org/) [23], in which related genes' PPPI data was extracted from *Saccharomyces* Genome Database (SGD, http://www.yeastgenome.org/) derived from BioGRID (http://thebiogrid.org/) with MiMIplugin software (http://apps.cytoscape.org/apps/mimiplugin) [24]. The PPPI networks obtained were analyzed with MCODE software (http://apps.cytoscape.org/apps/mcode) [25], [26] to detect clusters of proteins that represent distinct biologic processes. Major network centralities (node degree and betweenness) were calculated from these networks and clusters using the Cytoscape plugin CentiScaPe 1.0 (http://apps.cytoscape.org/apps/centiscape) [26], [27] to further identify the bottlenecks, which are nodes with values above the threshold and central points in controlling the communication between other nodes within the network [28]. Gene Ontology (GO) clustering analysis was performed using BiNGO software (http://apps.cytoscape.org/apps/bingo) [29] in order to obtain information about the nature and number of sub networks belonging to the network and their associated biological processes. Overrepresented biological process categories were generated after correction with a significance level of 0.05 [26].

### Annexin V and Propidium iodide staining

Annexin V and Propidium iodide (PI) staining were performed according to the published method [7], [30]. *S. cerevisiae* cells were washed with sorbitol buffer (1.2 M sorbitol, 0.5 mM MgCl_2_, 35 mM potassium phosphate, pH 6.8), incubated at 30°C for 15 min in Tris/DTT buffer (100 mM Tris/Cl, pH 9.4, 10 mM DTT), washed again with sorbitol buffer, and digested at 30°C for 1 h with Zymolyase-20T (MP Biomedicals) in sorbitol buffer. After protoplast formation was observed under light microscopy, cells were harvested, washed with binding buffer (10 mM HEPES, pH 7.4, 140 mM NaCl, 2.5 mM CaCl_2_, 1.2 M sorbitol), and stained using Annexin-V-Fluos Staining Kit (Roche Diagnostics). To prepare the labeling solution, 20 µL Annexin-V-Fluos labeling reagent was diluted in 1 mL binding buffer and 20 µL PI solutions was added. Around 10^6^ *S. cerevisiae* cells were centrifuged and resuspended in 100 µL Annexin-V-Fluos labeling solution. After incubation for 10∼15 min in dark at room temperature, 500 µL binding buffer was added for analysis using the BD flow cytometry (BD FACSCalibur, BD Biosciences). 488 nm excitation and 515 nm bandpass filter (FL1) for fluorescein detection and filter >560 nm (FL2) for PI detection were chosen. All assays were performed in triplicate. A mixture containing equal volumes of heat-treated cells and normal cells (50% heat-treated cells) were performed as positive control to assist the set of cross gate in the data analysis. The flow cytometry data were processed using Kaluza Flow Cytometry Software.

### Detection of metacaspase activity

Metacaspase activity was detected using FITC-VAD-FMK (Promega) [31], [32]. After LPS treatment, *S. cerevisiae* cells were washed and resuspended in PBS. 100 µL yeast cells (10^6^ cells) were incubated with 50 µM of FITC-VAD-FMK for 20 min at 30°C in darkness. Then cells were washed once, resuspended in PBS and subsequently analyzed by BD flow cytometer equipped with a 488-nm argon laser. The 525 nm emission signals were detected using the FL1 bandpass filter. The 100% heat-treated cells in PBS were chosen as positive control. Cells without dye addition were performed to set the gate for distinguish negative and positive groups in the data extraction. The flow cytometry data were processed using Kaluza Flow Cytometry Software.

### Analysis of mitochondrial membrane potential and reactive oxygen species

Mitochondrial membrane potential (ΔΨ_m_) was determined using Rhodamine 123 (Rh123) (Sigma-Aldrich) staining methods [33]. *S. cerevisiae* cells were washed twice with PBS, and 1 mL cell suspension (10^6^ cells) was added 1 µL of Rh123 from 50 mM stock solution in ethanol and incubated for 10 min in darkness, and then analyzed using the BD flow cytometry equipped with a 488-nm argon laser. The 525 nm emission signals were detected using the FL1 bandpass filter. The 100% heat-treated cells were chosen here as positive control, and corresponding cells without dye addition was performed to set the gate for negative and positive groups in the data extraction. The flow cytometry data were processed using Kaluza Flow Cytometry Software.

For detecting reactive oxygen species (ROS) production, 1 mL pre-log phase yeast cells (10^7^ cells) were incubated with 20 µM of the fluorescence probe, 2′,7′-dichlorofluorescin diacetate (H_2_DCFH-DA, Sigma-Aldrich) in YPD for 15 min at 30°C in darkness [8], [34]. The cells were then washed twice with PBS and treated with or without 1.0 mg/mL LPS in PBS (OD_600_ = 2.0) for 60 min at 30°C in darkness. The flow cytometry analysis was immediately carried out using the BD flow cytometry. 488 nm excitation and 520 nm emission by the FL1 bandpass filter was chosen for 2′,7′-dichlorofluorescein detection. Corresponding cells without dye loading were performed to set the gate for negative and positive groups in the data extraction. The flow cytometry data were processed using Kaluza Flow Cytometry Software.

## Results

### *S. cerevisiae* BY4742 cells could be stained by methylene blue after exposure to LPS

**Figure 1:**
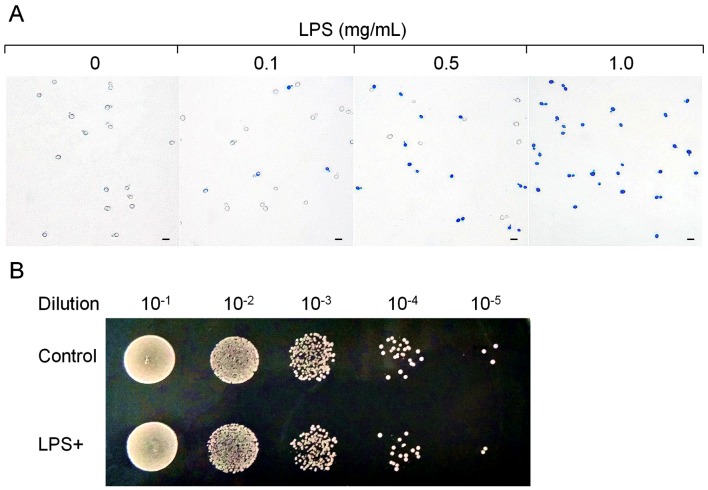
Effect of LPS stimualtion to *S. cerevisiae* BY4742. A. Methylene blue staining of *S. cerevisiae* BY4742 cells treated with different concentrations of LPS and observed using light microscopy. The solid bars in the photos indicate 10 µm. The concentrations of LPS were listed on top of the photos. B. Drop test showed that *S. cerevisiae* BY4742 cells treated with 1.0 mg mg/mL LPS were not dead.

Methylene blue, a heterocyclic aromatic chemical compound, can be used as an indicator to determine if yeast cells are alive [18], [35]. Viable yeast cells usually cannot be stained by methylene blue because their membranes cannot be penetrated. The dead or injured yeast cells can be stained because their membranes are damaged and cannot keep the methylene blue from penetrating. In this study, three different methylene blue solutions [18]–[20] were used to stain *S. cerevisiae* BY4742 cells. At the same methylene blue concentration (3.0 mg/mL), none of the three methylene blue solutions could stain *S. cerevisiae* BY4742 cells; however, all three methylene blue solutions could stain the LPS-treated BY4742 cells. The staining was pH independent because pH values of the three methylene blue solutions are different. Fig. 1A showed the microscopic pictures of *S. cerevisiae* BY4742 cells exposed to different concentrations of LPS and stained by Loeffler's alkaline methylene blue solution. The proportion of the cells that could be stained by methylene blue increased with the increase of LPS concentration in the mixture. After exposure to 1.0 mg/mL LPS, around 5×10^8^ *S. cerevisiae* cells in 1 mL mixture (OD_600_ = 2.0) could be all stained by methylene blue. The staining of LPS-treated *S. cerevisiae* BY4742 cells suggests that the cells were either dead or injured after LPS treatment because methylene blue could not penetrate normal cell's membrane.

To assess the viability of LPS-treated *S. cerevisiae* BY4742 cells, the cells were serially diluted and dropped on YPD plates, using the untreated cells as control (Fig. 1B). After 3-day incubation, colonies were observed on plates where the LPS-treated cells were dropped, and their numbers were similar to the untreated cells, suggesting that *S. cerevisiae* BY4742 cells were still alive after exposure to LPS. Moreover, when *S. cerevisiae* BY4742 cells were incubated in liquid SD media, they could keep growing; the proportion of the cells which could be stained by methylene blue kept stable until 90 min, and then decreased with time. This suggests again that *S. cerevisiae* BY4742 cells exposed to LPS were not dead but injured, but the injury can be repaired.

### LPS exposure caused significant changes in *S. cerevisiae* BY4742 cells

**Figure 2:**
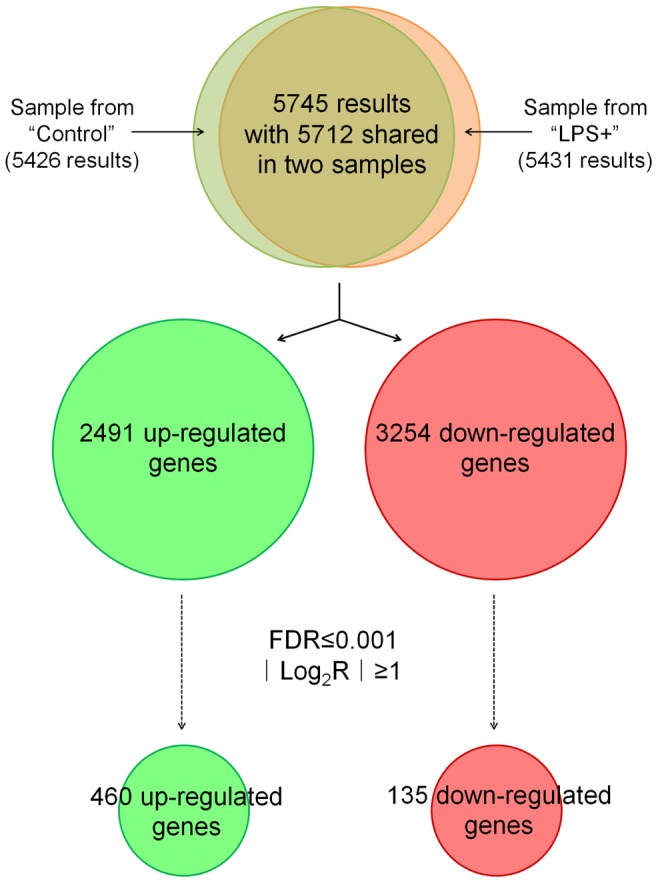
Number of differentially expressed genes regulated in *S. cerevisiae* BY4742 cells treated with LPS (LPS+) according to transcriptome analysis. FDR stands for False Discovery Rate [51]. Log_2_R represents Log2 of the ratio of the gene expression difference of a gene in “LPS+” samples versus the same gene in “Control” sample.

**Figure 3:**
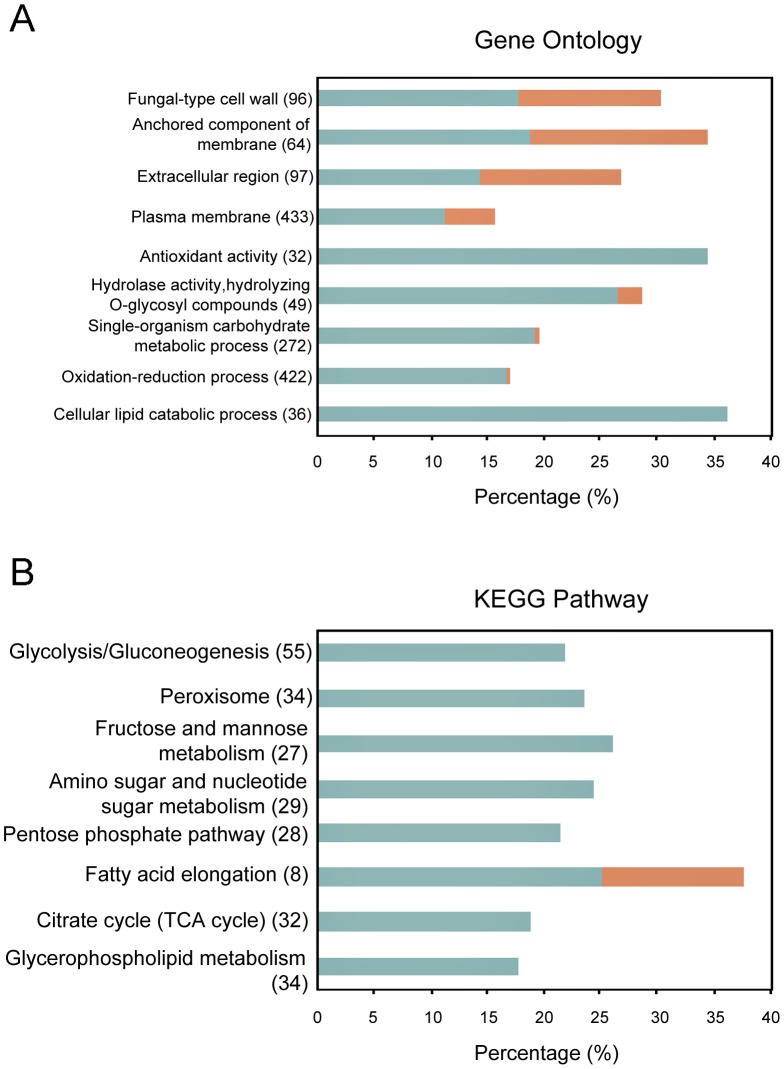
Analysis of Gene Ontology and KEGG pathway. Only the significantly enriched GO categories (A) or KEGG items (B) (*p*-value ≤0.05) in differentially expressed genes were shown. Blue and red bars represent up-regulated and down-regulated genes in LPS-treated cells compared to control, respectively. The percentage represents the ratio of genes within the genome in each category. The number of genes contained in each item is shown in brackets following the item names.

To gain the insight of LPS stimulation to *S. cerevisiae*, the transcriptional profling of the *S. cerevisiae* BY4742 cells with or without LPS treatment for 90 min were analyzed [22]. The transcriptome analysis detected 5431 and 5426 genes from *S. cerevisiae* BY4742 cells treated with or without LPS treatment, respectively. After exposure to LPS, 2491 genes were up-regulated in *S. cerevisiae* BY4742 cells and 3254 genes were down-regulated, and 595 genes (460 up-regulated genes and 135 down-regulated genes) were significantly modulated (FDR ≤0.001 and |Log_2_R| ≥1.0) (Fig. 2). GO analysis was performed to classify the main biological processes associated to these 595 genes. The probability values of the over-abundance of the GO groups compared to the genome average were assessed, and major GO groups with the statistical significance of overrepresented processes (*p*-value ≤0.05) are listed in Fig. 3. These major GO categories are closely related to cell wall, membrane, lipid, carbohydrate, and oxidation-reduction process. In the categories of antioxidant activity and lipid catabolic process, all related genes were up-regulated, while in categories of cell wall and membrane, both up-regulated and down-regulated genes were observed. These gene regulations related to cell wall and membrane might change the stability or permeability of the cell; therefore, methylene blue could penetrate and stain the cells (Fig. 1A). Regulations on cell wall, membrane, and antioxidant activity are usually related to stress response; therefore, transcriptional levels of genes in GO categories with less statistical significance (*p*-value >0.05) but closely related to stress responses are listed in Table S4. There are 69 genes involved in peroxisome, and 15 of them were up-regulated, suggesting that the function of peroxisome increased after LPS stimulation. Mitochondria were also affected after LPS stimulation, but more genes related to its outer membrane and intermembrane space were up-regulated than to its inner membrane (Table S4). Some genes related to protein folding, autophagy, endocytosis, and apoptosis were up-regulated after LPS stimulation, suggesting that cell injure might occur. Interestingly, 27 genes related to ribosome biogenesis were down-regulated after LPS stimulation, suggesting protein syntheses were repressed in the cells (Table S4). Autophagy usually involves degradation pathways including peroxisome degradation [10], while apoptosis always leads to cell death [3], [4]. Peroxisome and mitochondrial function are associated with antioxidant activity [36]. Apart from GO analysis, significantly enriched items of KEGG pathway also provided information about basal metabolism such as peroxisome metabolism (Fig. 3B). The data indicate that LPS stimulation caused a series of regulations in *S. cerevisiae* BY4742, involving cell wall, membrane, peroxisome and mitochondria.

**Figure 4:**
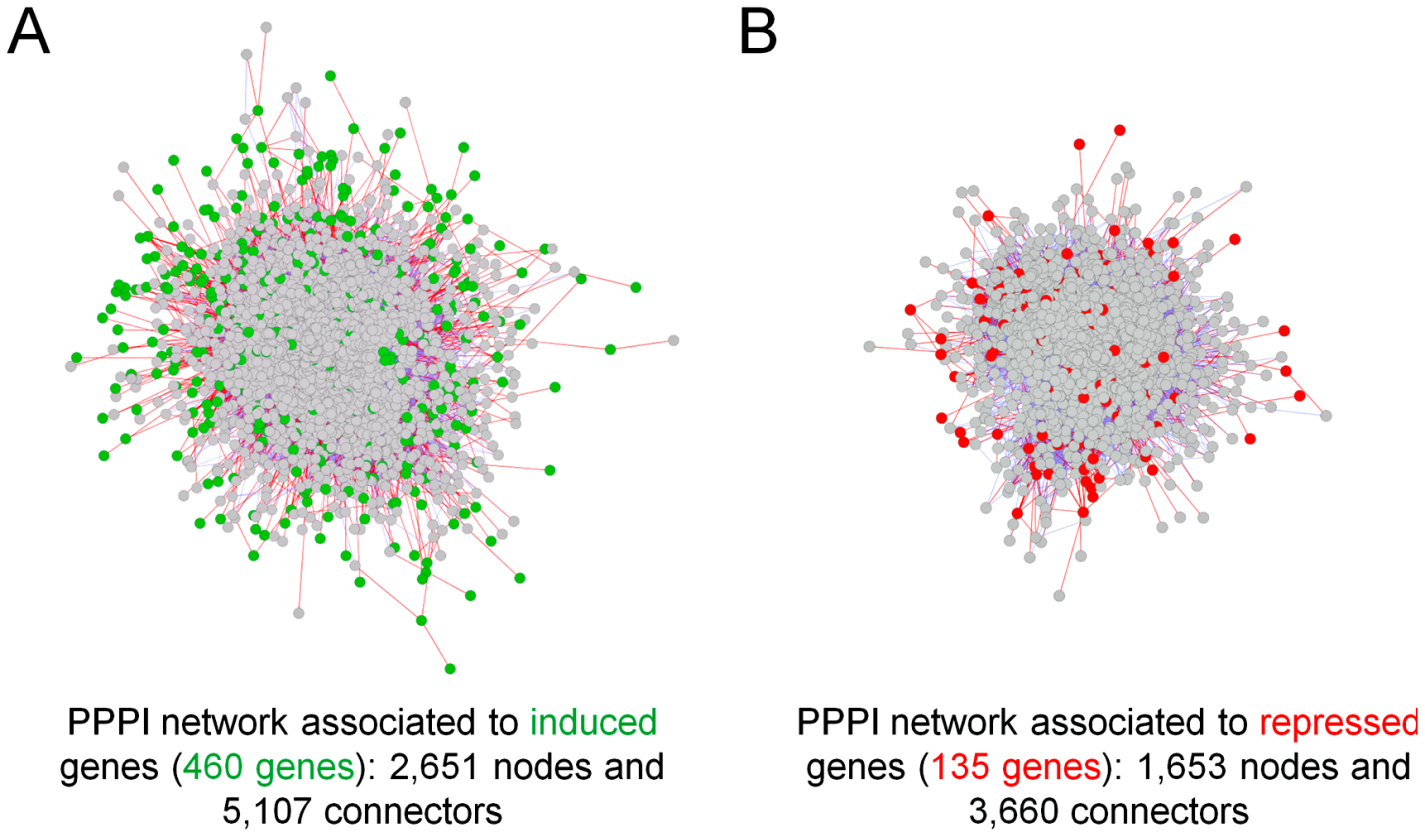
PPPI networks generated from the induced genes (A) and the repressed genes (B). Green nodes indicate proteins encoded by the induced genes, while red nodes represent proteins encoded by the repressed genes. Red lines indicate the interactions among proteins encoded by differentially expressed genes.

To understand how LPS stimulation affects biological processes in *S. cerevisiae* BY4742, two PPPI networks were retrieved from SGD using the transcriptome data: one associated to the 460 induced genes (367 genes were picked up) and the other associated to the 135 repressed genes (108 genes were picked up) (Fig. 4). The induced genes-associated network contains 2,651 nodes and 5,107 connectors (Fig. 4A), and the repressed genes-associated network contains 1,653 nodes and 3,660 connectors (Fig. 4B). Nodes represent proteins encoded by the corresponding genes in the network, and connectors are introduced to represent interactions among the proteins encoded by the differentially expressed genes. Clusters present in both PPPI networks were identified and retrieved using MCODE, and subjected to GO analysis; there were 8 clusters extracted from the induced genes-associated network (Table S1) and 11 clusters obtained from the repressed genes-associated network (Table S2). Moreover, there were 23 biological processes associated to more than 10 clusters (Table 1), including response to stress, signal transduction, chromosome, transcription, ribosome biogenesis, enzyme regulator activity and cell cycle. This suggests that LPS stimulation caused a series of responses in different biological processes from chromosome segregation to protein modification process. To identify nodes and the consequent biological processes that have a relevant important position in the overall network architecture, the centrality analysis of both networks and their clusters were performed using CentiScaPe; there were 26 and 27 bottlenecks, the key nodes with high values of nodes degree and betweeness, identified from the induced and repressed genes-associated networks, respectively (Table S3).

**Table 1:** Major specific gene ontology categories shared by more than 10 clusters derived from the induced and repressed genes-associated networks.

Description	Total cluster numbers	Induced genes related	repressed genes related
response to stress	12	5	7
signal transduction	12	5	7
			
chromosome	14	5	9
chromosome organization	16	7	9
chromosome segregation	12	4	8
helicase activity	10	1	9
nucleus	16	6	10
nucleolus	12	4	8
DNA metabolic process	12	4	8
DNA binding	14	5	9
			
transcription	17	8	9
transcription regulator activity	15	6	9
RNA metabolic process	16	6	10
ribosome biogenesis	12	4	8
			
cell cycle	14	6	8
cytoskeleton	13	7	6
cytoskeleton organization	14	7	7
endoplasmic reticulum	12	7	5
endomembrane system	11	8	3
			
protein modification process	16	7	9
protein complex biogenesis	10	5	5
protein binding	13	6	7
enzyme regulator activity	10	3	7

**Figure 5:**
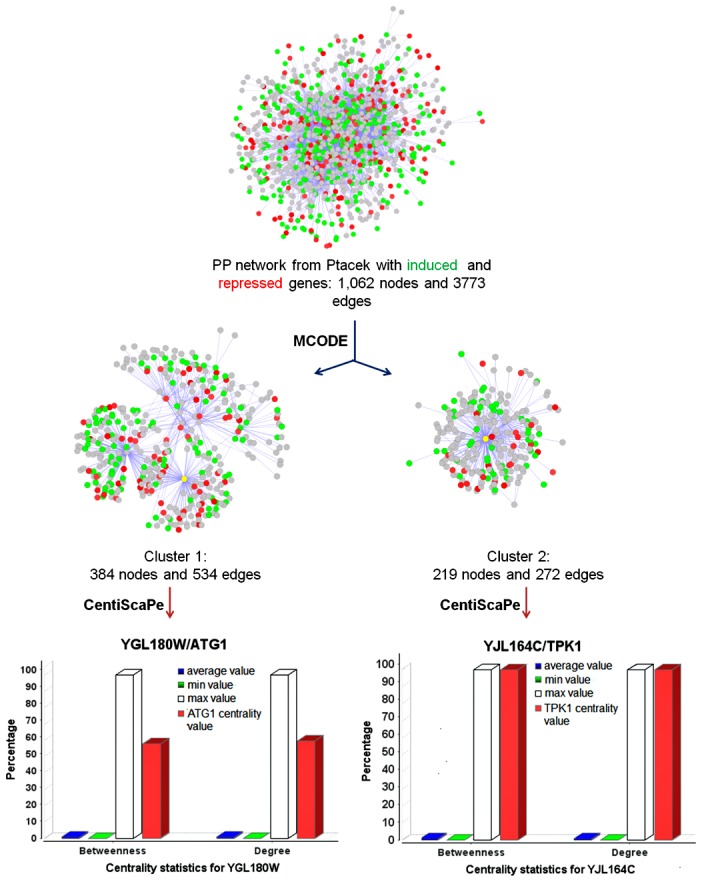
Protein phosphorylation network generated from the induced genes and the repressed genes, and its MCODE and CentiScaPe analyses. Green nodes indicate proteins encoded by the induced genes, while red nodes represent proteins encoded by the repressed genes. Deep colored nodes are associated to genes with |Log_2_R| ≥1.0 while light colored nodes are associated to genes with 0.5≤ |Log_2_R| ≤1.0. Yellow nodes indicate Atg1p and Tpk1p in Cluster1 and Cluster 2, respectively. Corresponding centrality values in betweeness and nodes degree were sub graphed according to the importance of selected nodes or bottlenecks. Edges represent interactions between proteins or nodes.

Since there are over 4,000 phosphorylation events involved in yeast [37], changes in protein phosphorylation (PP) network of *S. cerevisiae* BY4742 cells after exposure to LPS might provide new information on the mechanism. Therefore, the PP network was analyzed using more gene expression data (|Log_2_R| ≥0.5) and cluster analysis was performed as before (Fig. 5). Two clusters (Cluster 1 and Cluster 2) were sub graphed, and their central bottlenecks were *ATG1* and *TPK1,* respectively (Fig. 5). *ATG1* encodes a kinase required for vesicle formation in autophagy and the cytoplasm-to-vacuole targeting pathway, while *TPK1* encodes the catalytic subunit of the cAMP-dependent protein kinase that promotes vegetative growth in response to nutrients via the Ras-cAMP signaling pathway (SGD, Table S3). These two protein phosphorylation signaling pathways provide possible activated points during the cell exposure to LPS.

### RT-PCR of some key genes in *S. cerevisiae* BY4742 and methylene blue staining of *S. cerevisiae* BY4743 mutants after LPS treatment

**Figure 6:**
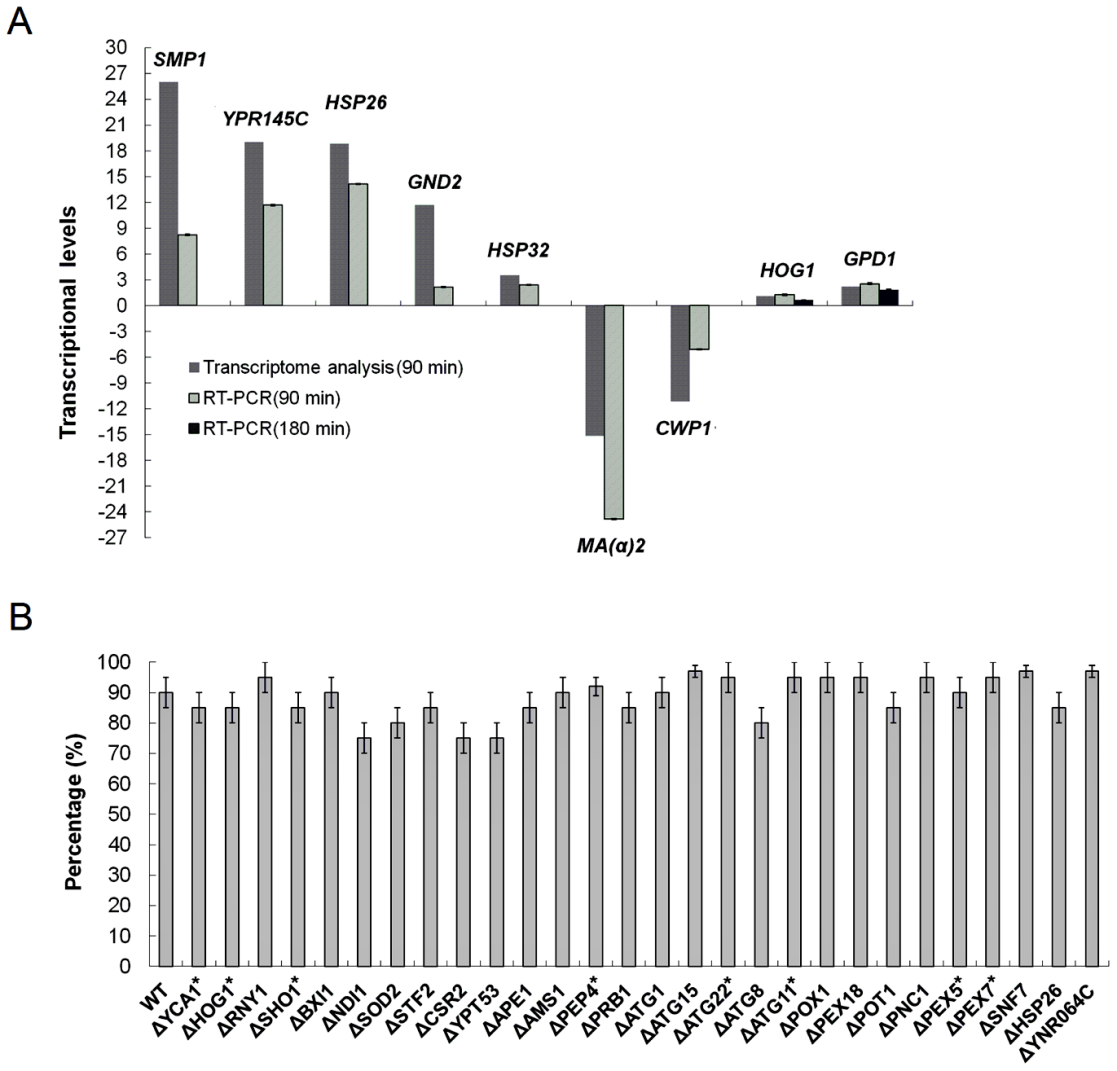
A. Comparison of transcriptional levels of some key genes in LPS-treated *S. cerevisiae* BY4742 cells analyzed by RT-PCR and transcriptome, respectively. B. Methylene blue staining of some single gene deletion mutants of *S. cerevisiae* BY4743 after LPS treatment. According to the transcriptome analysis, genes whose transcriptional level did not significantly change in *S. cerevisiae* BY4742 after LPS treatment were labeled with star symbol (*); transcriptional levels for the other genes could be found in Table S4.

To confirm the regulation information obtained by the transcriptome analysis, a few key genes were chosen to be analyzed by RT-PCR. The overall results from RT-PCR are consistent with those obtained by transcriptome analysis. In both RT-PCR and transcriptome analysis, genes *SMP1, YPR145C HSP26, GND2, HSP32* and *GPD1* were found up-regulated, while genes *MA(α)2* and *CWP1* were down-regulated (Fig. 6A). The transcriptional levels of a gene might depend on the time of cell growth and LPS treatment. For example, the transcriptional levels of *HOG1* and *GPD1* in *S. cerevisiae* BY4742 cells treated with LPS for 90 min were larger than those treated with LPS for 180 min (Fig. 6A).

To investigate the mechanism of the response of *S. cerevisiae* to LPS stimulation, some mutants of *S. cerevisiae* BY4743 were treated with LPS and stained by methylene blue (Fig. 6B). These mutants are all single gene deletion ones. These genes, including apoptosis or autophagy related genes *YCA1*, *NDI1*, *ATG1* and *ATG8*, were chosen based on the transcriptome analysis of *S. cerevisiae* BY4742 treated with LPS. Surprisingly, after treatment with LPS most of the mutants could be stained by methylene blue, suggesting that the response of *S. cerevisiae* to LPS stimulation is a complex process, and individually deletion of these genes could not stop the response. For example, *ATG1* mutant of *S. cerevisiae* BY4743 treated with LPS could be stained by methylene blue although *ATG1* is one of the major bottlenecks in PP network.

### LPS treatment caused phosphatidylserine exposure and mitochondrial membrane potential increase in *S. cerevisiae* BY4742 cells

**Figure 7:**
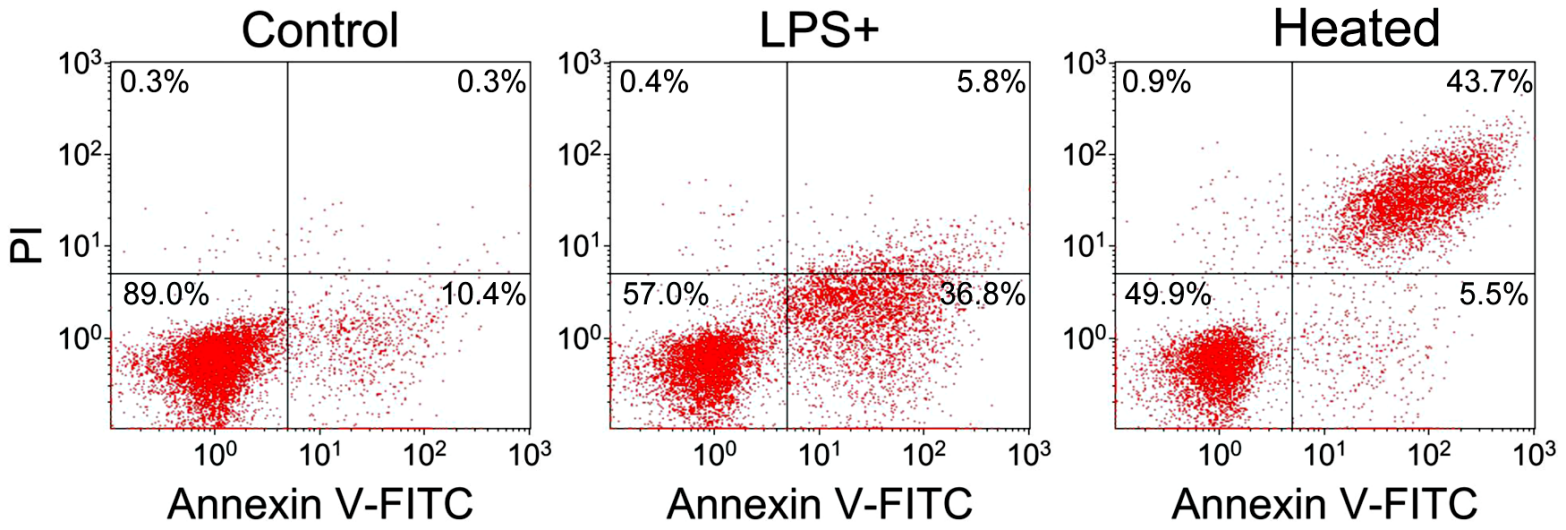
Flow cytometry analysis of LPS-treated *S. cerevisiae* BY4742 cells stained by Annexin-V-FITC and PI. Untreated cells were used as blank control (Control) and 50% heat-treated dead cells were used as positive control (Heated). The cross gate, in order to show the stained proportions, was set by both blank control and positive control.

All cells are separated from the extracellular environment by the membrane, a phospholipid bilayer. In eukaryotic cells, the lipid composition of the outer and inner leaflets of the membrane is not symmetrical. Phosphatidylserine is restricted to the inner leaflet of the membrane, and the exposure of phosphatidylserine to the outer leaflet of the membrane is a classic feature of apoptotic cells and acts as an “eat me” signal allowing phagocytosis [3], [30]. Since the transcriptome analysis showed that many modulated genes in *S. cerevisiae* BY4742 cells treated with LPS were related to cell response (Table S4), FITC-labeled Annexin V was used to detect the exposure of phosphatidylserine in the cells [30], using 50% heat-treated dead cells as positive control [38]. About 57.0% population of LPS-treated *S. cerevisiae* BY4742 cells showed similar pattern to the major population of untreated cells (89.0%), but 36.8% population of LPS-treated cells showed different patterns either from the untreated cells or the heat-treated dead cells (Fig. 7), suggesting that this 36.8% population of LPS-treated *S. cerevisiae* BY4742 cells were injured but not dead. The membrane integrity was tested by staining with propidium iodide [30]. Propidium iodide could be taken up by 43.7% of the heat-treated cells, but only by 5.8% of the LPS-treated cells and 0.3% of the untreated *S. cerevisiae* BY4742 cells, respectively (Fig. 7). This suggests that after LPS stimulation the cell membranes of *S. cerevisiae* BY4742 might be slightly injured for signal transduction but not damaged as that of the heat-treated cells, and also explains the methylene blue staining and the viability of *S. cerevisiae* BY4742 cells after exposure to LPS.

**Figure 8:**
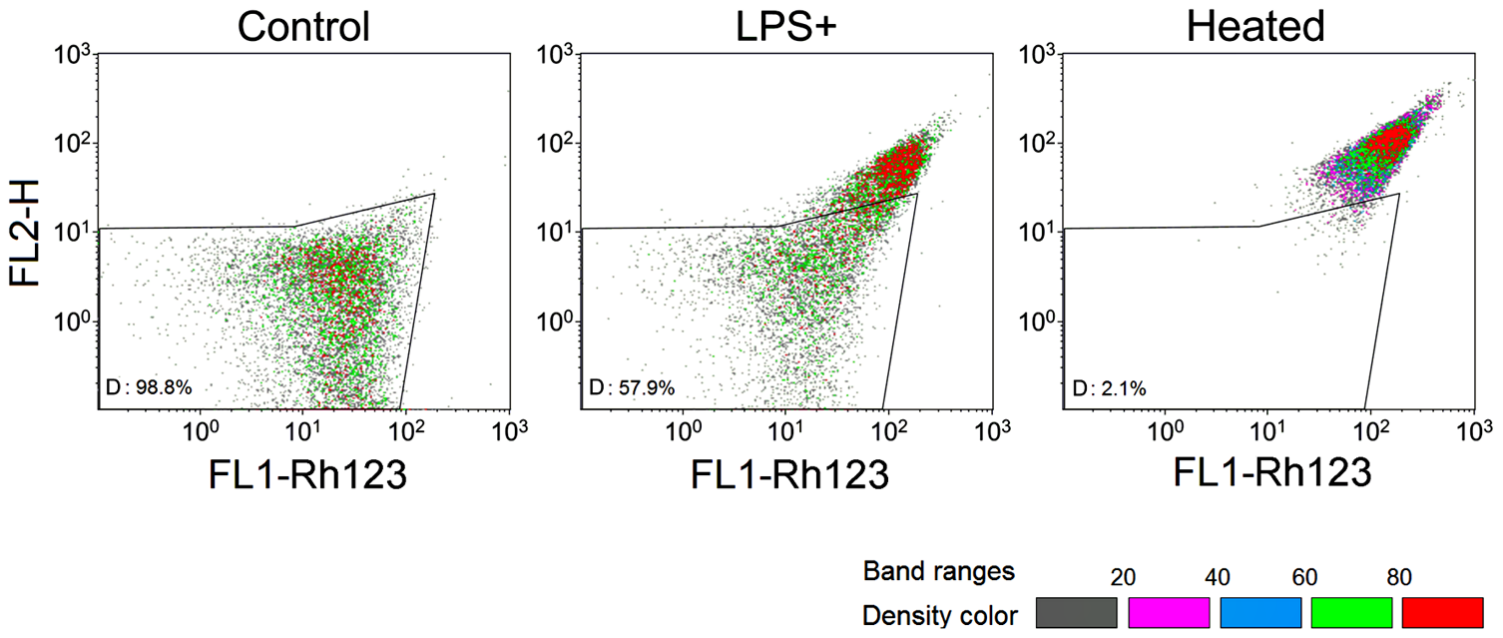
Assessment of mitochondrial membrane potential in LPS-treated *S. cerevisiae* BY4742 cells, using flow cytometry. Rh123 was used to stain the untreated (Control), LPS-treated (LPS+) and 100% heat-treated cells (Heated). The result was represented by density banding coloring. Gray dots represent the lowest density, while red dots represent the highest density. The gate set “G” was used to show the differences among the three experiments.

Since mitochondria are preferential targets for various stresses in *S. cerevisiae* [3], mitochondrial function or integrity in *S. cerevisiae* BY4742 cells treated with LPS was assessed by staining with Rh123 [33]. Rh123 can distribute into the mitochondrial matrix in response to ΔΨ_m_. The untreated cells and heat-treated *S. cerevisiae* cells were used as the negative and positive controls, respectively. As shown in Fig. 8, about half of the LPS-treated *S. cerevisiae* BY4742 cells had similar ΔΨ_m_ to the heat-treated cells. This increase of ΔΨ_m_ in LPS-treated *S. cerevisiae* cells might be due to many factors such as the inhibition of ATP synthase [36], [39].

### LPS treatment did not affect the ROS level and metacaspase activation in *S. cerevisiae* BY4742 cells

**Figure 9:**
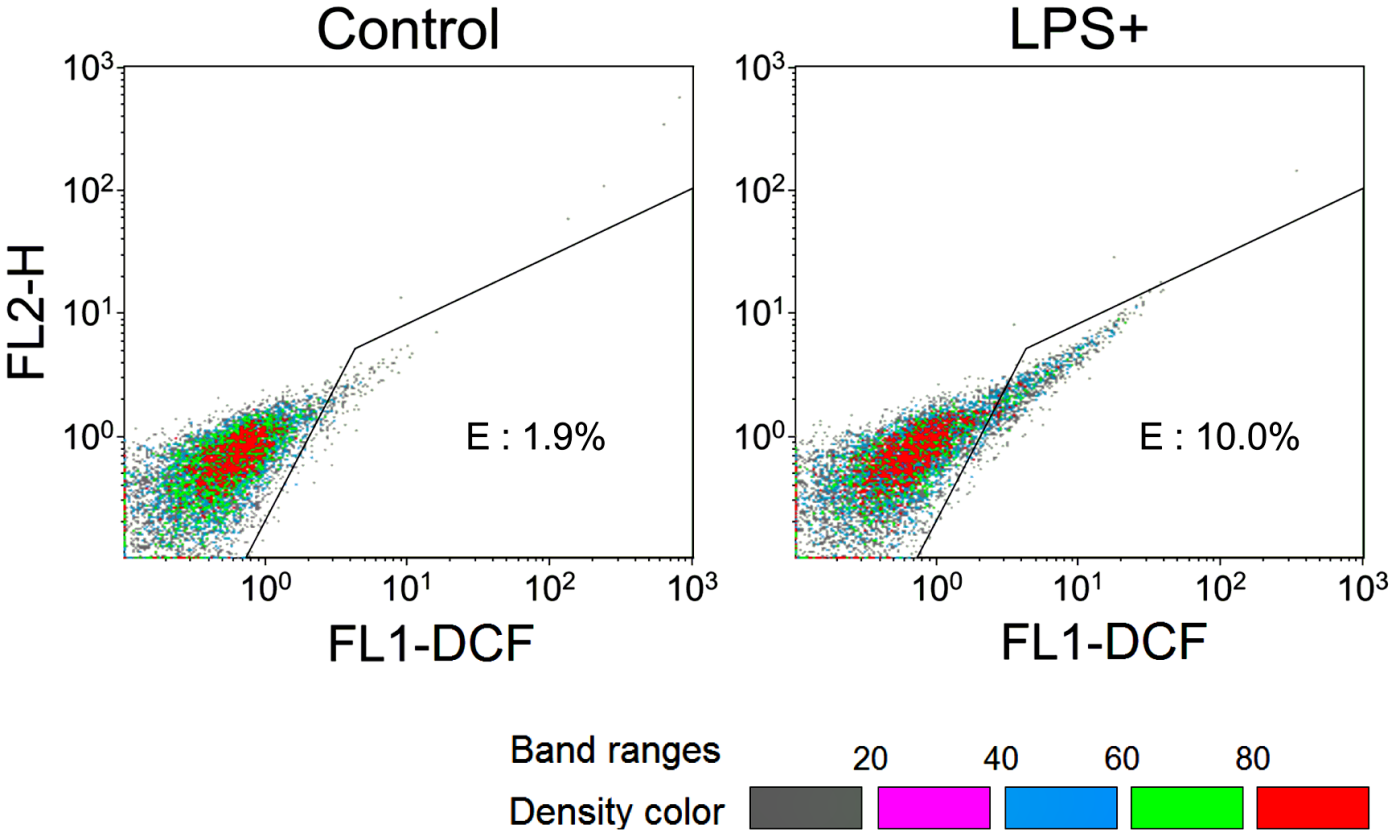
Detection of ROS accumulation in LPS-treated *S. cerevisiae* BY4742 cells, using flow cytometry. H_2_DCFH-DA was used to stain the untreated (Control) and LPS-treated cells (LPS+). The result was represented by density banding coloring. Gray dots represent the lowest density, while red dots represent the highest density. The gate “E” was set by corresponding dye-unloading samples. FL1-DCF represents 2′,7′-dichlorofluorescein while FL2-H assists for data display. The majority populations (red dots) from both samples shew similar ROS levels.

ROS level in LPS-treated *S. cerevisiae* BY4742 cells was analyzed by staining with H_2_DCFH-DA [34]. H_2_DCFH-DA can be hydrolyzed by esterase to produce 2′,7′-dichlorofluorescin, which is then oxidized by ROS to form the fluorescent 2′,7′-dichlorofluorescein. The ROS levels in the majority of LPS-treated *S. cerevisiae* BY4742 cells were similar to the untreated cells (Fig. 9), indicating the ROS stability in LPS-treated *S. cerevisiae* BY4742 cells.

**Figure 10:**
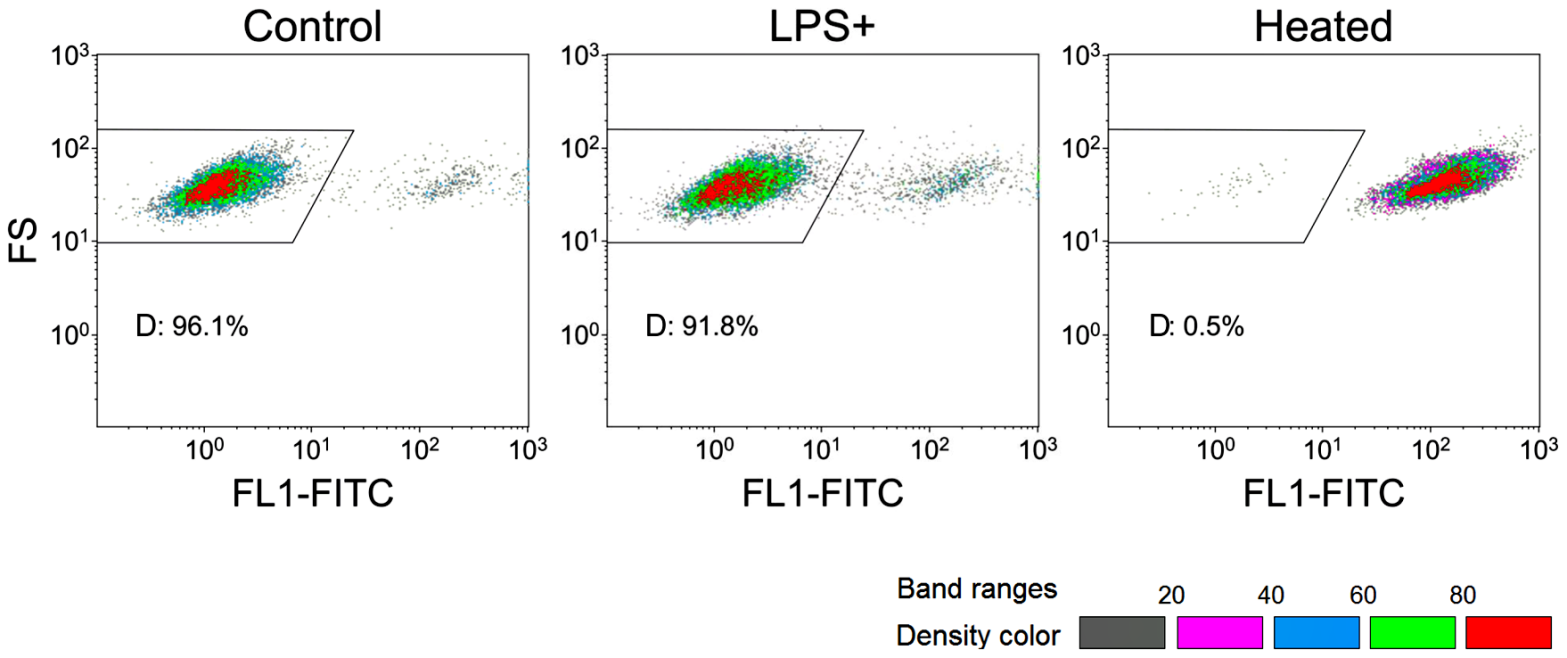
Analysis of metacaspase activity of LPS-treated *S. cerevisiae* BY4742 cells by flow cytometry. FITC-VAD-FMK was used to detect metacaspace activation. Untreated *S. cerevisiae* BY4742 cells were used as negative control; 100% heat-treated cells were chosen as positive control. The result was represented by density banding coloring. Gray dots represent the lowest density, while red dots represent the highest density. The gate “D” was set to show the differences among the three types of cells. FL1-FITC represents FITC-VAD-FMK while FS assists for data display.

Metacaspase activation in LPS-treated *S. cerevisiae* BY4742 cells was detected by staining with FITC-VAD-FMK and analyzed by flow cytometry [31], [32]. The population distribution pattern of LPS-treated *S. cerevisiae* BY4742 cells was similar to the untreated cells (Fig. 10), suggesting the metacaspase activation in *S. cerevisiae* BY4742 cells was not affected by LPS treatment. This is consistent with the finding in transcriptome analysis that the transcriptional level of *YCA1*, the key gene encoding metacaspase in *S. cerevisiae* BY4742 cells, did not significantly change after LPS treatment [40].

## Discussion

To survive under different stresses in the environment, microorganisms have developed a variety of mechanisms to give specific and adaptive responses. In response to stresses, *S. cerevisiae* could regulate different biological processes in the cell [6]–[8], [41], [42], involving phosphatidylserine, mitochondrial membrane, ROS and nucleus [3], [43]. For example, pheromone stress can induce phosphatidylserine externalization and cause the hyperpolarization of mitochondrial membrane (the elevation of ΔΨ_m_), the ROS production, and then the fragmentation of mitochondria (the decrease of ΔΨ_m_) and the release of cytochrome c to finally induce cell death [41]. The decreased ΔΨ_m_ and increased ROS production in yeast were also observed in response to aspirin, rapamycin, osmotin or formic acid [7], [8], [44], [45].

Phosphatidylserine exposure and enhanced ΔΨ_m_ were observed in LPS-treated *S. cerevisiae* cells, but ROS production and metacaspase activation did not change (Fig. 7–10). This indicates that LPS stimulation did change some biological processes, but did not cause cell death. Activated antioxidant activity (Fig. 3) might be the reason for the stability of ROS, while the repressed ribosome biogenesis (Table S4) could save energy to be used for removing harmful substances. Enhanced mitochondrial function should be important for energy production and ROS removal, associated to the exchange with peroxisomes undergoing β-oxidation for ROS transport or energy reserve. Because ROS stability was controlled, LPS-treated *S. cerevisiae* cells could survive and be recovered after LPS removal or dilution. Autophagy process might occur in LPS-treated *S. cerevisiae* cells, because 10 *ATGs* (Table S4) were up-regulated after LPS stimulation, and Atg1p, the protein kinase required for vesicle formation in autophagy, was a central point in both the PP network and the induced genes-associated PPPI network (Fig. 5 and Table S3). Various protein folding or degradation underwent at the same time, such as assisted by chaperones via vesicles, to renovate the intracellular environment for survival (Table S4). More information about the differentially expressed genes in LPS-treated *S. cerevisiae* cells is listed in Table S5.

Since yeast cells live in the same environment with other microorganisms including LPS-containing bacteria, they might have evolved mechanisms to sense and adapt to the presence of LPS. Response of *S. cerevisiae* to LPS found in this study might be important for investigating yeast evolution to cope with the environment. Because other microorganisms could recognize and interact with LPS [46], [47], LPS-containing bacteria have also evolved mechanisms to change their LPS structure [48]–[50]. Further work should be done to answer the following questions: Which parts of LPS can stimulate yeast? Is there a sensor in the membrane of yeast to recognize LPS? What is the signal transduction pathway in yeast cells after LPS stimulation? Such study might contribute to understand the LPS recognition by other eukaryotic cells.

## Supporting Information

Table S1 **Main specific gene ontology categories observed in clusters (MCODE Score >3.0) derived from the induced genes-associated network.**
(DOC)

Table S2 **Main specific gene ontology categories observed in clusters (MCODE Score >3.0) derived from the repressed genes-associated network.**
(DOC)

Table S3 **Selected bottleneck nodes with different gene expression (|Log_2_R| ≥1.0) observed in networks.**
(DOC)

Table S4 **Details of genes extracted from Gene Ontology analysis and their classification from the description in SGD.**
(DOC)

Table S5 **Other Genes with the expression ratio of Log2 more than 2 or less than -0.5 found in LPS-treated** ***S. cerevisiae*** **BY4742 cells.**
(DOC)
