# Supplementary material for: Response of Saccharomyces cerevisiae to the Stimulation of Lipopolysaccharide
Source: PLoS One. 2014 Aug 8;9(8):e104428. doi: 10.1371/journal.pone.0104428 (PMC4126697; doi:10.1371/journal.pone.0104428)
Supplement: Table S1 — Main specific gene ontology categories observed in clusters (MCODE Score >3.0) derived from the induced genes-associated network. (DOC) [file pone.0104428.s001.doc]

**Table S1. Main specific gene ontology categories observed in clusters (MCODE Score >3.0) derived from the induced genes-associated network.**

|  | Description | GO-ID | Corrected *P*-value*^a^* | x*^b^*/n*^c^* |
| --- | --- | --- | --- | --- |
| Cluster 1 | chromosome organization | 0051276 | 2.39E-66 | 187/408 |
|  | transcription | 0006350 | 1.69E-53 | 206/563 |
|  | nucleus | 0005634 | 2.76E-50 | 457/2121 |
|  | chromosome | 0005694 | 1.00E-32 | 134/371 |
|  | DNA metabolic process | 0006259 | 9.54E-32 | 151/460 |
|  | transcription regulator activity | 0030528 | 2.10E-25 | 120/361 |
|  | cell cycle | 0007049 | 1.17E-23 | 164/608 |
|  | response to stress | 0006950 | 9.29E-20 | 172/704 |
|  | protein modification process | 0006464 | 1.95E-19 | 160/639 |
|  | chromosome segregation | 0007059 | 7.84E-17 | 64/168 |
|  | DNA binding | 0003677 | 1.63E-16 | 141/570 |
|  | meiosis | 0007126 | 1.44E-13 | 62/183 |
|  | RNA metabolic process | 0016070 | 8.04E-11 | 168/837 |
|  | protein complex biogenesis | 0070271 | 3.51E-09 | 65/242 |
|  | protein binding | 0005515 | 6.85E-07 | 131/688 |
|  | cellular protein catabolic process | 0044257 | 2.24E-05 | 65/301 |
|  | cytoskeleton organization | 0007010 | 8.67E-05 | 50/222 |
|  | signal transduction | 0007165 | 9.15E-05 | 56/259 |
|  | motor activity | 0003774 | 4.38E-04 | 11/25 |
|  | cytoskeleton | 0005856 | 1.07E-03 | 50/245 |
|  | Golgi apparatus | 0005794 | 1.43E-03 | 49/242 |
|  | sporulation resulting in formation of a cellular spore | 0030435 | 1.88E-03 | 45/220 |
|  | endoplasmic reticulum | 0005783 | 2.63E-03 | 79/448 |
|  | conjugation | 0000746 | 4.24E-03 | 27/118 |
|  | ligase activity | 0016874 | 9.33E-03 | 35/174 |
|  | endomembrane system | 0012505 | 9.35E-03 | 93/568 |
|  | cell budding | 0007114 | 2.90E-02 | 19/86 |
|  | helicase activity | 0004386 | 3.81E-02 | 22/107 |
| Cluster 2 | chromosome organization | 0051276 | 1.04E-48 | 159/408 |
|  | transcription | 0006350 | 1.98E-33 | 167/563 |
|  | nucleus | 0005634 | 6.76E-31 | 390/2121 |
|  | chromosome | 0005694 | 2.05E-25 | 117/371 |
|  | cell cycle | 0007049 | 4.63E-23 | 155/608 |
|  | protein modification process | 0006464 | 1.80E-18 | 150/639 |
|  | protein complex biogenesis | 0070271 | 1.57E-14 | 73/242 |
|  | DNA metabolic process | 0006259 | 6.69E-14 | 110/460 |
|  | chromosome segregation | 0007059 | 2.85E-13 | 56/168 |
|  | transcription regulator activity | 0030528 | 1.29E-11 | 88/361 |
|  | DNA binding | 0003677 | 2.15E-09 | 115/570 |
|  | response to stress | 0006950 | 6.63E-09 | 133/704 |
|  | cellular protein catabolic process | 0044257 | 5.37E-08 | 69/301 |
|  | cytoskeleton organization | 0007010 | 1.07E-07 | 55/222 |
|  | RNA metabolic process | 0016070 | 6.47E-07 | 144/837 |
|  | meiosis | 0007126 | 9.22E-07 | 46/183 |
|  | protein binding | 0005515 | 1.27E-06 | 122/688 |
|  | cytoskeleton | 0005856 | 1.56E-05 | 53/245 |
|  | endomembrane system | 0012505 | 2.01E-04 | 96/568 |
|  | motor activity | 0003774 | 2.01E-04 | 11/25 |
|  | nucleus organization | 0006997 | 3.52E-04 | 20/70 |
|  | vesicle-mediated transport | 0016192 | 3.56E-04 | 67/370 |
|  | Golgi apparatus | 0005794 | 7.65E-04 | 47/242 |
|  | microtubule organizing center | 0005815 | 2.85E-03 | 18/70 |
|  | peroxisome organization | 0007031 | 3.48E-03 | 16/60 |
|  | transport | 0006810 | 3.63E-03 | 173/1221 |
|  | signal transduction | 0007165 | 9.98E-03 | 45/259 |
|  | peroxisome | 0005777 | 2.75E-02 | 14/61 |
|  | ligase activity | 0016874 | 2.75E-02 | 31/174 |
|  | sporulation resulting in formation of a cellular spore | 0030435 | 3.44E-02 | 37/220 |
|  | cellular membrane organization | 0016044 | 4.65E-02 | 43/269 |
| Cluster 3 | chromosome organization | 0051276 | 2.20E-36 | 145/408 |
|  | transcription | 0006350 | 6.41E-27 | 159/563 |
|  | nucleus | 0005634 | 4.70E-25 | 386/2121 |
|  | cell cycle | 0007049 | 2.24E-16 | 144/608 |
|  | chromosome | 0005694 | 1.54E-14 | 99/371 |
|  | Golgi apparatus | 0005794 | 1.25E-13 | 73/242 |
|  | endomembrane system | 0012505 | 3.20E-13 | 129/568 |
|  | endoplasmic reticulum | 0005783 | 5.42E-12 | 106/448 |
|  | vesicle-mediated transport | 0016192 | 1.77E-10 | 89/370 |
|  | protein modification process | 0006464 | 4.05E-10 | 131/639 |
|  | RNA metabolic process | 0016070 | 1.13E-09 | 159/837 |
|  | transcription regulator activity | 0030528 | 1.30E-09 | 85/361 |
|  | chromosome segregation | 0007059 | 6.67E-08 | 47/168 |
|  | DNA metabolic process | 0006259 | 6.76E-08 | 96/460 |
|  | protein complex biogenesis | 0070271 | 2.07E-07 | 59/242 |
|  | vesicle organization | 0016050 | 9.20E-07 | 26/74 |
|  | cellular protein catabolic process | 0044257 | 9.44E-07 | 67/301 |
|  | ribosome biogenesis | 0042254 | 2.49E-06 | 77/372 |
|  | protein binding | 0005515 | 2.61E-06 | 124/688 |
|  | cytoskeleton | 0005856 | 3.51E-06 | 56/245 |
|  | meiosis | 0007126 | 5.12E-06 | 45/183 |
|  | response to stress | 0006950 | 8.03E-06 | 124/704 |
|  | cytoskeleton organization | 0007010 | 8.23E-06 | 51/222 |
|  | DNA binding | 0003677 | 1.97E-05 | 103/570 |
|  | microtubule organizing center | 0005815 | 4.27E-05 | 22/70 |
|  | cytoplasmic membrane-bounded vesicle | 0016023 | 1.59E-04 | 28/108 |
|  | nucleolus | 0005730 | 5.30E-04 | 53/271 |
|  | sporulation resulting in formation of a cellular spore | 0030435 | 5.50E-04 | 45/220 |
|  | enzyme regulator activity | 0030234 | 5.50E-04 | 49/246 |
|  | cellular membrane organization | 0016044 | 7.44E-04 | 52/269 |
|  | motor activity | 0003774 | 9.97E-04 | 10/25 |
|  | transport | 0006810 | 1.83E-03 | 180/1221 |
|  | structural molecule activity | 0005198 | 4.66E-03 | 64/375 |
|  | conjugation | 0000746 | 7.52E-03 | 25/118 |
|  | nucleus organization | 0006997 | 1.89E-02 | 16/70 |
|  | cell cortex | 0005938 | 2.86E-02 | 25/131 |
| Cluster 4 | endoplasmic reticulum | 0005783 | 8.16E-21 | 136/448 |
|  | transcription | 0006350 | 7.09E-17 | 149/563 |
|  | endomembrane system | 0012505 | 1.24E-14 | 144/568 |
|  | chromosome organization | 0051276 | 2.17E-13 | 111/408 |
|  | nucleus | 0005634 | 8.95E-13 | 380/2121 |
|  | Golgi apparatus | 0005794 | 1.25E-11 | 74/242 |
|  | protein modification process | 0006464 | 1.31E-09 | 141/639 |
|  | protein binding | 0005515 | 1.90E-08 | 145/688 |
|  | RNA metabolic process | 0016070 | 3.27E-08 | 168/837 |
|  | protein complex biogenesis | 0070271 | 8.03E-08 | 65/242 |
|  | cytoskeleton organization | 0007010 | 5.52E-07 | 59/222 |
|  | vesicle-mediated transport | 0016192 | 2.05E-06 | 84/370 |
|  | cellular protein catabolic process | 0044257 | 7.85E-06 | 70/301 |
|  | transcription regulator activity | 0030528 | 1.91E-05 | 79/361 |
|  | cytoskeleton | 0005856 | 3.26E-05 | 58/245 |
|  | cell cycle | 0007049 | 3.74E-05 | 118/608 |
|  | cell cortex | 0005938 | 6.07E-05 | 36/131 |
|  | vesicle organization | 0016050 | 8.73E-05 | 24/74 |
|  | response to stress | 0006950 | 2.17E-04 | 129/704 |
|  | cytoplasmic membrane-bounded vesicle | 0016023 | 2.20E-04 | 30/108 |
|  | nucleolus | 0005730 | 2.81E-04 | 59/271 |
|  | chromosome | 0005694 | 6.84E-04 | 74/371 |
|  | cellular membrane organization | 0016044 | 7.37E-04 | 57/269 |
|  | signal transduction | 0007165 | 8.63E-04 | 55/259 |
|  | transport | 0006810 | 1.97E-03 | 199/1221 |
|  | cellular lipid metabolic process | 0044255 | 3.24E-03 | 50/243 |
|  | membrane | 0016020 | 3.42E-03 | 286/1852 |
|  | protein folding | 0006457 | 4.94E-03 | 27/113 |
|  | site of polarized growth | 0030427 | 5.16E-03 | 48/237 |
|  | cellular bud | 0005933 | 5.16E-03 | 42/201 |
|  | enzyme regulator activity | 0030234 | 6.19E-03 | 49/246 |
|  | ribosome biogenesis | 0042254 | 6.19E-03 | 69/372 |
|  | cellular cell wall organization | 0007047 | 6.59E-03 | 50/254 |
|  | cell wall organization | 0071555 | 6.59E-03 | 50/254 |
|  | hydrolase activity | 0016787 | 6.59E-03 | 151/923 |
|  | DNA metabolic process | 0006259 | 7.07E-03 | 82/460 |
|  | motor activity | 0003774 | 8.69E-03 | 9/25 |
|  | cell budding | 0007114 | 8.87E-03 | 21/86 |
|  | chromosome segregation | 0007059 | 9.80E-03 | 35/168 |
|  | DNA binding | 0003677 | 1.63E-02 | 96/570 |
|  | cytokinesis | 0000910 | 1.80E-02 | 24/109 |
|  | conjugation | 0000746 | 4.58E-02 | 24/118 |
| Cluster 5 | endoplasmic reticulum | 0005783 | 5.45E-16 | 138/448 |
|  | transcription | 0006350 | 2.41E-11 | 149/563 |
|  | endomembrane system | 0012505 | 3.48E-11 | 149/568 |
|  | nucleus | 0005634 | 1.22E-08 | 409/2121 |
|  | RNA metabolic process | 0016070 | 4.78E-08 | 188/837 |
|  | transport | 0006810 | 2.13E-06 | 247/1221 |
|  | Golgi apparatus | 0005794 | 2.13E-06 | 68/242 |
|  | cytoskeleton organization | 0007010 | 3.67E-06 | 63/222 |
|  | chromosome organization | 0051276 | 3.71E-06 | 100/408 |
|  | protein modification process | 0006464 | 3.71E-06 | 143/639 |
|  | vesicle-mediated transport | 0016192 | 6.12E-05 | 88/370 |
|  | transcription regulator activity | 0030528 | 2.19E-04 | 84/361 |
|  | nucleolus | 0005730 | 6.46E-04 | 65/271 |
|  | cell cortex | 0005938 | 6.54E-04 | 37/131 |
|  | protein binding | 0005515 | 6.72E-04 | 140/688 |
|  | membrane | 0016020 | 8.58E-04 | 332/1852 |
|  | cytoplasmic membrane-bounded vesicle | 0016023 | 1.41E-03 | 31/108 |
|  | cell cycle | 0007049 | 1.98E-03 | 123/608 |
|  | protein complex biogenesis | 0070271 | 6.10E-03 | 55/242 |
|  | cellular lipid metabolic process | 0044255 | 6.41E-03 | 55/243 |
|  | cellular protein catabolic process | 0044257 | 8.39E-03 | 65/301 |
|  | cellular membrane organization | 0016044 | 8.76E-03 | 59/269 |
|  | cytoskeleton | 0005856 | 3.00E-02 | 52/245 |
|  | cell budding | 0007114 | 3.34E-02 | 22/86 |
|  | motor activity | 0003774 | 3.34E-02 | 9/25 |
|  | protein folding | 0006457 | 3.76E-02 | 27/113 |
|  | ribosome biogenesis | 0042254 | 4.29E-02 | 73/372 |
|  | ligase activity | 0016874 | 4.29E-02 | 38/174 |
|  | vesicle organization | 0016050 | 4.35E-02 | 19/74 |
|  | cellular carbohydrate metabolic process | 0044262 | 4.35E-02 | 59/293 |
| Cluster 6 | transcription | 0006350 | 1.27E-14 | 130/563 |
|  | nucleus | 0005634 | 1.27E-14 | 343/2121 |
|  | endoplasmic reticulum | 0005783 | 3.60E-13 | 107/448 |
|  | endomembrane system | 0012505 | 3.31E-10 | 118/568 |
|  | RNA metabolic process | 0016070 | 2.49E-07 | 147/837 |
|  | plasma membrane | 0005886 | 3.06E-07 | 78/366 |
|  | membrane | 0016020 | 2.06E-05 | 268/1852 |
|  | cellular carbohydrate metabolic process | 0044262 | 2.06E-05 | 61/293 |
|  | membrane fraction | 0005624 | 2.11E-05 | 48/212 |
|  | transcription regulator activity | 0030528 | 2.11E-05 | 71/361 |
|  | DNA binding | 0003677 | 2.17E-05 | 101/570 |
|  | protein modification process | 0006464 | 4.87E-05 | 109/639 |
|  | cell cycle | 0007049 | 1.94E-04 | 102/608 |
|  | signal transduction | 0007165 | 2.02E-04 | 52/259 |
|  | cell cortex | 0005938 | 3.42E-04 | 31/131 |
|  | transport | 0006810 | 5.72E-04 | 179/1221 |
|  | site of polarized growth | 0030427 | 1.10E-03 | 46/237 |
|  | chromosome organization | 0051276 | 1.44E-03 | 70/408 |
|  | nucleolus | 0005730 | 1.89E-03 | 50/271 |
|  | cell budding | 0007114 | 2.54E-03 | 21/86 |
|  | cytoskeleton organization | 0007010 | 2.91E-03 | 42/222 |
|  | cellular lipid metabolic process | 0044255 | 2.91E-03 | 45/243 |
|  | pseudohyphal growth | 0007124 | 4.60E-03 | 18/73 |
|  | cellular bud | 0005933 | 4.60E-03 | 38/201 |
|  | cellular protein catabolic process | 0044257 | 5.38E-03 | 52/301 |
|  | cytoskeleton | 0005856 | 5.39E-03 | 44/245 |
|  | enzyme regulator activity | 0030234 | 5.64E-03 | 44/246 |
|  | ribosome biogenesis | 0042254 | 1.15E-02 | 60/372 |
|  | cellular cell wall organization | 0007047 | 1.64E-02 | 43/254 |
|  | cell wall organization | 0071555 | 1.64E-02 | 43/254 |
|  | transferase activity | 0016740 | 2.05E-02 | 108/755 |
|  | transporter activity | 0005215 | 3.17E-02 | 60/392 |
|  | response to stress | 0006950 | 4.28E-02 | 99/704 |
|  | chromosome | 0005694 | 4.86E-02 | 56/371 |
| Cluster 7 | endoplasmic reticulum | 0005783 | 9.30E-23 | 55/448 |
|  | endomembrane system | 0012505 | 5.87E-13 | 48/568 |
|  | transcription | 0006350 | 1.79E-09 | 42/563 |
|  | protein modification process | 0006464 | 7.61E-07 | 40/639 |
|  | membrane | 0016020 | 9.47E-07 | 79/1852 |
|  | Golgi apparatus | 0005794 | 1.39E-04 | 19/242 |
|  | cellular carbohydrate metabolic process | 0044262 | 5.38E-04 | 20/293 |
|  | chromosome organization | 0051276 | 2.36E-03 | 23/408 |
|  | cytoplasmic membrane-bounded vesicle | 0016023 | 3.51E-03 | 10/108 |
|  | transferase activity | 0016740 | 4.39E-03 | 34/755 |
|  | vesicle-mediated transport | 0016192 | 7.68E-03 | 20/370 |
|  | cellular lipid metabolic process | 0044255 | 8.37E-03 | 15/243 |
|  | transport | 0006810 | 8.69E-03 | 47/1221 |
| Cluster 8 | endoplasmic reticulum | 0005783 | 1.34E-28 | 116/448 |
|  | membrane | 0016020 | 2.94E-26 | 273/1852 |
|  | transport | 0006810 | 1.04E-23 | 202/1221 |
|  | vesicle-mediated transport | 0016192 | 4.08E-20 | 90/370 |
|  | endomembrane system | 0012505 | 4.08E-20 | 117/568 |
|  | protein binding | 0005515 | 3.39E-14 | 119/688 |
|  | Golgi apparatus | 0005794 | 1.42E-12 | 58/242 |
|  | vesicle organization | 0016050 | 4.54E-12 | 29/74 |
|  | cytoplasmic membrane-bounded vesicle | 0016023 | 5.72E-11 | 34/108 |
|  | peroxisome organization | 0007031 | 1.76E-09 | 23/60 |
|  | peroxisome | 0005777 | 2.37E-09 | 23/61 |
|  | site of polarized growth | 0030427 | 1.96E-08 | 49/237 |
|  | cellular membrane organization | 0016044 | 2.37E-08 | 53/269 |
|  | plasma membrane | 0005886 | 2.87E-08 | 65/366 |
|  | cytoplasm | 0005737 | 2.45E-07 | 403/4074 |
|  | cellular bud | 0005933 | 1.36E-06 | 40/201 |
|  | cell cortex | 0005938 | 1.88E-06 | 30/131 |
|  | cellular lipid metabolic process | 0044255 | 1.88E-06 | 45/243 |
|  | cell budding | 0007114 | 2.24E-06 | 23/86 |
|  | cytokinesis | 0000910 | 1.59E-04 | 23/109 |
|  | vacuole | 0005773 | 1.02E-03 | 37/239 |
|  | cytoskeleton organization | 0007010 | 1.02E-03 | 35/222 |
|  | membrane fraction | 0005624 | 1.85E-03 | 33/212 |
|  | cellular protein catabolic process | 0044257 | 3.35E-03 | 42/301 |
|  | signal transduction | 0007165 | 4.20E-03 | 37/259 |
|  | cytoskeleton | 0005856 | 5.45E-03 | 35/245 |
|  | cellular cell wall organization | 0007047 | 9.43E-03 | 35/254 |
|  | cell wall organization | 0071555 | 9.43E-03 | 35/254 |
|  | lipid binding | 0008289 | 1.27E-02 | 15/83 |
|  | sporulation resulting in formation of a cellular spore | 0030435 | 3.58E-02 | 29/220 |
|  | transcription | 0006350 | 3.95E-02 | 63/563 |
|  | nucleus organization | 0006997 | 4.14E-02 | 12/70 |

*^a^* Calculated values based on *P* values that calculated by the hypergeometric distribution of one ontology class visualized in the network obtained after FDR was applied.

*^b^* Total number of proteins found in the network which belong to a gene ontology.

*^c^* Total number of proteins that belong to a specific gene ontology.
