# Supplementary material for: Response of Saccharomyces cerevisiae to the Stimulation of Lipopolysaccharide
Source: PLoS One. 2014 Aug 8;9(8):e104428. doi: 10.1371/journal.pone.0104428 (PMC4126697; doi:10.1371/journal.pone.0104428)
Supplement: Table S2 — Main specific gene ontology categories observed in clusters (MCODE Score >3.0) derived from the repressed genes-associated network. (DOC) [file pone.0104428.s002.doc]

**Table S2. Main specific gene ontology categories observed in clusters (MCODE Score >3.0) derived from the repressed genes-associated network.**

|  | Description | GO-ID | Corrected *P*-value*^a^* | x*^b^*/n*^c^* |
| --- | --- | --- | --- | --- |
| Cluster 1 | ribosome biogenesis | 0042254 | 0.00E+00 | 160/372 |
|  | nucleolus | 0005730 | 3.66E-81 | 110/271 |
|  | ribosome | 0005840 | 2.92E-71 | 103/275 |
|  | structural molecule activity | 0005198 | 6.59E-55 | 102/375 |
|  | RNA metabolic process | 0016070 | 6.63E-53 | 143/837 |
|  | RNA binding | 0003723 | 1.93E-32 | 113/776 |
|  | translation | 0006412 | 1.11E-22 | 111/959 |
|  | nucleus | 0005634 | 5.32E-16 | 163/2121 |
|  | helicase activity | 0004386 | 2.69E-03 | 14/107 |
| Cluster 2 | nucleus | 0005634 | 7.85E-86 | 392/2121 |
|  | nucleolus | 0005730 | 6.73E-62 | 123/271 |
|  | ribosome biogenesis | 0042254 | 1.43E-60 | 142/372 |
|  | chromosome organization | 0051276 | 1.65E-49 | 136/408 |
|  | RNA metabolic process | 0016070 | 9.11E-47 | 196/837 |
|  | chromosome | 0005694 | 9.96E-42 | 120/371 |
|  | DNA metabolic process | 0006259 | 2.44E-38 | 130/460 |
|  | cell cycle | 0007049 | 4.86E-26 | 132/608 |
|  | response to stress | 0006950 | 7.45E-18 | 128/704 |
|  | chromosome segregation | 0007059 | 1.23E-16 | 52/168 |
|  | transcription | 0006350 | 1.23E-16 | 108/563 |
|  | DNA binding | 0003677 | 7.30E-12 | 98/570 |
|  | helicase activity | 0004386 | 5.88E-10 | 32/107 |
|  | meiosis | 0007126 | 1.89E-09 | 43/183 |
|  | ribosome | 0005840 | 6.44E-07 | 50/275 |
|  | RNA binding | 0003723 | 8.18E-06 | 102/776 |
|  | structural molecule activity | 0005198 | 1.56E-05 | 58/375 |
|  | transcription regulator activity | 0030528 | 7.12E-04 | 51/361 |
|  | protein complex biogenesis | 0070271 | 2.47E-03 | 36/242 |
|  | protein modification process | 0006464 | 4.50E-03 | 76/639 |
|  | hydrolase activity | 0016787 | 7.50E-03 | 102/923 |
|  | signal transduction | 0007165 | 7.50E-03 | 36/259 |
|  | cytoskeleton organization | 0007010 | 4.55E-02 | 29/222 |
| Cluster 3 | nucleus | 0005634 | 1.14E-86 | 522/2121 |
|  | chromosome organization | 0051276 | 8.21E-67 | 188/408 |
|  | nucleolus | 0005730 | 1.18E-53 | 137/271 |
|  | chromosome | 0005694 | 6.28E-53 | 162/371 |
|  | RNA metabolic process | 0016070 | 5.52E-42 | 242/837 |
|  | ribosome biogenesis | 0042254 | 2.50E-41 | 147/372 |
|  | transcription | 0006350 | 5.92E-37 | 181/563 |
|  | cell cycle | 0007049 | 5.62E-32 | 181/608 |
|  | DNA metabolic process | 0006259 | 3.66E-29 | 147/460 |
|  | chromosome segregation | 0007059 | 2.69E-22 | 72/168 |
|  | DNA binding | 0003677 | 2.83E-18 | 146/570 |
|  | response to stress | 0006950 | 8.85E-15 | 160/704 |
|  | transcription regulator activity | 0030528 | 1.23E-11 | 93/361 |
|  | protein modification process | 0006464 | 1.33E-09 | 134/639 |
|  | cytoskeleton | 0005856 | 3.09E-09 | 66/245 |
|  | protein complex biogenesis | 0070271 | 1.22E-08 | 64/242 |
|  | helicase activity | 0004386 | 5.70E-08 | 36/107 |
|  | cytoskeleton organization | 0007010 | 9.45E-06 | 53/222 |
|  | signal transduction | 0007165 | 5.33E-05 | 57/259 |
|  | meiosis | 0007126 | 5.33E-05 | 44/183 |
|  | protein binding | 0005515 | 6.20E-05 | 123/688 |
|  | microtubule organizing center | 0005815 | 3.67E-04 | 21/70 |
|  | motor activity | 0003774 | 3.94E-04 | 11/25 |
|  | enzyme regulator activity | 0030234 | 6.45E-04 | 51/246 |
|  | cell budding | 0007114 | 1.03E-03 | 23/86 |
|  | nucleotidyltransferase activity | 0016779 | 5.90E-03 | 28/126 |
|  | conjugation | 0000746 | 9.29E-03 | 26/118 |
|  | cellular bud | 0005933 | 1.98E-02 | 38/201 |
|  | protein folding | 0006457 | 3.85E-02 | 23/113 |
|  | site of polarized growth | 0030427 | 3.85E-02 | 42/237 |
|  | cytokinesis | 0000910 | 4.69E-02 | 22/109 |
| Cluster 4 | nucleus | 0005634 | 1.09E-69 | 439/2121 |
|  | chromosome organization | 0051276 | 7.55E-56 | 161/408 |
|  | chromosome | 0005694 | 4.76E-46 | 141/371 |
|  | transcription | 0006350 | 3.34E-34 | 160/563 |
|  | cell cycle | 0007049 | 9.26E-29 | 158/608 |
|  | nucleolus | 0005730 | 4.75E-23 | 89/271 |
|  | chromosome segregation | 0007059 | 1.18E-22 | 67/168 |
|  | RNA metabolic process | 0016070 | 4.91E-22 | 179/837 |
|  | DNA metabolic process | 0006259 | 5.13E-19 | 115/460 |
|  | ribosome biogenesis | 0042254 | 9.20E-17 | 96/372 |
|  | DNA binding | 0003677 | 2.41E-15 | 124/570 |
|  | cytoskeleton | 0005856 | 6.09E-12 | 65/245 |
|  | transcription regulator activity | 0030528 | 1.62E-11 | 83/361 |
|  | cytoskeleton organization | 0007010 | 6.09E-10 | 57/222 |
|  | protein modification process | 0006464 | 1.59E-09 | 118/639 |
|  | protein complex biogenesis | 0070271 | 2.29E-09 | 59/242 |
|  | response to stress | 0006950 | 5.26E-09 | 125/704 |
|  | microtubule organizing center | 0005815 | 9.90E-08 | 25/70 |
|  | conjugation | 0000746 | 5.73E-06 | 31/118 |
|  | helicase activity | 0004386 | 6.00E-05 | 27/107 |
|  | protein binding | 0005515 | 7.46E-05 | 107/688 |
|  | structural molecule activity | 0005198 | 7.97E-05 | 66/375 |
|  | meiosis | 0007126 | 1.36E-04 | 38/183 |
|  | nucleotidyltransferase activity | 0016779 | 4.13E-04 | 28/126 |
|  | cellular protein catabolic process | 0044257 | 4.73E-04 | 53/301 |
|  | signal transduction | 0007165 | 1.03E-03 | 46/259 |
|  | motor activity | 0003774 | 2.41E-03 | 9/25 |
|  | nucleus organization | 0006997 | 7.58E-03 | 16/70 |
|  | hydrolase activity | 0016787 | 3.46E-02 | 119/923 |
|  | enzyme regulator activity | 0030234 | 3.46E-02 | 38/246 |
|  | protein folding | 0006457 | 4.47E-02 | 20/113 |
| Cluster 5 | nucleus | 0005634 | 6.23E-46 | 332/2121 |
|  | transcription | 0006350 | 6.15E-44 | 153/563 |
|  | chromosome organization | 0051276 | 2.66E-37 | 120/408 |
|  | RNA metabolic process | 0016070 | 1.24E-32 | 172/837 |
|  | chromosome | 0005694 | 2.46E-23 | 94/371 |
|  | DNA binding | 0003677 | 6.52E-20 | 115/570 |
|  | cell cycle | 0007049 | 1.18E-16 | 113/608 |
|  | DNA metabolic process | 0006259 | 1.81E-15 | 92/460 |
|  | transcription regulator activity | 0030528 | 5.20E-15 | 78/361 |
|  | nucleolus | 0005730 | 8.12E-15 | 65/271 |
|  | nucleotidyltransferase activity | 0016779 | 5.35E-12 | 38/126 |
|  | ribosome biogenesis | 0042254 | 2.49E-08 | 65/372 |
|  | chromosome segregation | 0007059 | 1.66E-07 | 37/168 |
|  | cytoskeleton | 0005856 | 1.54E-06 | 45/245 |
|  | response to stress | 0006950 | 3.51E-06 | 95/704 |
|  | helicase activity | 0004386 | 8.41E-06 | 25/107 |
|  | protein complex biogenesis | 0070271 | 1.54E-05 | 42/242 |
|  | protein modification process | 0006464 | 4.34E-05 | 84/639 |
|  | cytoskeleton organization | 0007010 | 7.26E-04 | 35/222 |
|  | motor activity | 0003774 | 2.99E-03 | 8/25 |
|  | signal transduction | 0007165 | 3.16E-03 | 37/259 |
|  | enzyme regulator activity | 0030234 | 4.49E-03 | 35/246 |
|  | microtubule organizing center | 0005815 | 6.28E-03 | 14/70 |
|  | transferase activity | 0016740 | 9.91E-03 | 84/755 |
|  | protein binding | 0005515 | 1.24E-02 | 77/688 |
|  | cell cortex | 0005938 | 4.07E-02 | 19/131 |
| Cluster 6 | endoplasmic reticulum | 0005783 | 5.68E-35 | 45/448 |
|  | membrane | 0016020 | 1.61E-28 | 63/1852 |
|  | endomembrane system | 0012505 | 7.62E-27 | 42/568 |
|  | transport | 0006810 | 8.07E-08 | 34/1221 |
|  | cellular lipid metabolic process | 0044255 | 2.32E-07 | 15/243 |
|  | vesicle-mediated transport | 0016192 | 2.48E-07 | 18/370 |
|  | protein modification process | 0006464 | 1.58E-04 | 19/639 |
|  | cytoplasm | 0005737 | 2.16E-04 | 58/4074 |
|  | transporter activity | 0005215 | 1.12E-03 | 13/392 |
|  | Golgi apparatus | 0005794 | 4.84E-03 | 9/242 |
|  | protein folding | 0006457 | 5.52E-03 | 6/113 |
|  | transferase activity | 0016740 | 7.67E-03 | 17/755 |
|  | vacuole | 0005773 | 1.40E-02 | 8/239 |
|  | plasma membrane | 0005886 | 1.78E-02 | 10/366 |
|  | vesicle organization | 0016050 | 2.61E-02 | 4/74 |
| Cluster 7 | chromosome organization | 0051276 | 3.24E-38 | 128/408 |
|  | cell cycle | 0007049 | 2.26E-31 | 149/608 |
|  | transcription | 0006350 | 4.10E-30 | 140/563 |
|  | chromosome | 0005694 | 5.14E-29 | 108/371 |
|  | nucleus | 0005634 | 1.12E-27 | 318/2121 |
|  | cytoskeleton | 0005856 | 1.08E-13 | 63/245 |
|  | transcription regulator activity | 0030528 | 1.41E-12 | 78/361 |
|  | chromosome segregation | 0007059 | 4.09E-11 | 46/168 |
|  | cytoskeleton organization | 0007010 | 7.75E-11 | 54/222 |
|  | DNA metabolic process | 0006259 | 8.15E-11 | 87/460 |
|  | DNA binding | 0003677 | 8.15E-11 | 101/570 |
|  | site of polarized growth | 0030427 | 8.15E-11 | 56/237 |
|  | cellular bud | 0005933 | 1.39E-10 | 50/201 |
|  | microtubule organizing center | 0005815 | 5.41E-09 | 25/70 |
|  | response to stress | 0006950 | 3.01E-07 | 106/704 |
|  | cell cortex | 0005938 | 2.84E-06 | 31/131 |
|  | protein modification process | 0006464 | 1.85E-05 | 92/639 |
|  | protein binding | 0005515 | 2.32E-05 | 97/688 |
|  | RNA metabolic process | 0016070 | 2.63E-05 | 113/837 |
|  | cell budding | 0007114 | 2.73E-05 | 22/86 |
|  | enzyme regulator activity | 0030234 | 3.85E-05 | 44/246 |
|  | signal transduction | 0007165 | 6.30E-05 | 45/259 |
|  | sporulation resulting in formation of a cellular spore | 0030435 | 3.19E-04 | 38/220 |
|  | conjugation | 0000746 | 5.13E-04 | 24/118 |
|  | helicase activity | 0004386 | 7.89E-04 | 22/107 |
|  | protein folding | 0006457 | 1.70E-03 | 22/113 |
|  | meiosis | 0007126 | 1.49E-02 | 28/183 |
|  | endoplasmic reticulum | 0005783 | 1.80E-02 | 57/448 |
|  | motor activity | 0003774 | 1.80E-02 | 7/25 |
|  | protein complex biogenesis | 0070271 | 2.19E-02 | 34/242 |
|  | pseudohyphal growth | 0007124 | 4.13E-02 | 13/73 |
| Cluster 8 | nucleus | 0005634 | 3.88E-20 | 95/2121 |
|  | RNA metabolic process | 0016070 | 1.01E-18 | 59/837 |
|  | ribosome biogenesis | 0042254 | 1.09E-14 | 36/372 |
|  | transcription | 0006350 | 1.00E-12 | 41/563 |
|  | nucleolus | 0005730 | 2.03E-10 | 26/271 |
|  | chromosome organization | 0051276 | 6.55E-08 | 28/408 |
|  | transcription regulator activity | 0030528 | 3.63E-07 | 25/361 |
|  | chromosome | 0005694 | 1.19E-04 | 21/371 |
|  | helicase activity | 0004386 | 2.25E-03 | 9/107 |
|  | DNA binding | 0003677 | 1.48E-02 | 22/570 |
| Cluster 9 | transcription | 0006350 | 9.76E-21 | 80/563 |
|  | chromosome organization | 0051276 | 9.98E-21 | 67/408 |
|  | nucleus | 0005634 | 3.32E-11 | 150/2121 |
|  | chromosome | 0005694 | 1.82E-09 | 46/371 |
|  | RNA metabolic process | 0016070 | 3.73E-06 | 68/837 |
|  | transcription regulator activity | 0030528 | 4.31E-05 | 36/361 |
|  | DNA metabolic process | 0006259 | 5.15E-05 | 42/460 |
|  | endoplasmic reticulum | 0005783 | 1.36E-04 | 40/448 |
|  | protein modification process | 0006464 | 6.94E-04 | 49/639 |
|  | enzyme regulator activity | 0030234 | 6.94E-04 | 25/246 |
|  | site of polarized growth | 0030427 | 9.32E-04 | 24/237 |
|  | cellular bud | 0005933 | 1.49E-03 | 21/201 |
|  | response to stress | 0006950 | 1.49E-03 | 51/704 |
|  | protein binding | 0005515 | 1.49E-03 | 50/688 |
|  | chromosome segregation | 0007059 | 2.51E-03 | 18/168 |
|  | cell cycle | 0007049 | 3.58E-03 | 44/608 |
|  | conjugation | 0000746 | 1.08E-02 | 13/118 |
|  | DNA binding | 0003677 | 1.84E-02 | 39/570 |
|  | protein folding | 0006457 | 1.93E-02 | 12/113 |
|  | endomembrane system | 0012505 | 2.71E-02 | 38/568 |
| Cluster 10 | chromosome organization | 0051276 | 1.26E-18 | 93/408 |
|  | nucleus | 0005634 | 3.70E-16 | 269/2121 |
|  | chromosome | 0005694 | 1.13E-13 | 78/371 |
|  | transcription | 0006350 | 1.05E-10 | 95/563 |
|  | cell cycle | 0007049 | 5.89E-10 | 98/608 |
|  | RNA metabolic process | 0016070 | 2.39E-08 | 118/837 |
|  | site of polarized growth | 0030427 | 1.67E-07 | 47/237 |
|  | nucleolus | 0005730 | 1.55E-06 | 49/271 |
|  | ribosome biogenesis | 0042254 | 1.78E-05 | 58/372 |
|  | cytoskeleton | 0005856 | 4.30E-05 | 42/245 |
|  | cellular bud | 0005933 | 6.96E-05 | 36/201 |
|  | cytoskeleton organization | 0007010 | 1.07E-04 | 38/222 |
|  | DNA binding | 0003677 | 1.27E-04 | 76/570 |
|  | chromosome segregation | 0007059 | 1.27E-04 | 31/168 |
|  | protein binding | 0005515 | 2.09E-04 | 87/688 |
|  | DNA metabolic process | 0006259 | 2.65E-04 | 63/460 |
|  | transcription regulator activity | 0030528 | 3.19E-04 | 52/361 |
|  | enzyme regulator activity | 0030234 | 3.31E-04 | 39/246 |
|  | protein modification process | 0006464 | 5.10E-04 | 80/639 |
|  | cell cortex | 0005938 | 8.64E-04 | 24/131 |
|  | vesicle-mediated transport | 0016192 | 1.77E-03 | 50/370 |
|  | helicase activity | 0004386 | 2.02E-03 | 20/107 |
|  | microtubule organizing center | 0005815 | 6.78E-03 | 14/70 |
|  | endoplasmic reticulum | 0005783 | 8.26E-03 | 55/448 |
|  | cell budding | 0007114 | 1.75E-02 | 15/86 |
|  | response to stress | 0006950 | 1.75E-02 | 78/704 |
|  | signal transduction | 0007165 | 1.83E-02 | 34/259 |
|  | protein folding | 0006457 | 1.87E-02 | 18/113 |
|  | conjugation | 0000746 | 2.84E-02 | 18/118 |
| Cluster 11 | chromosome organization | 0051276 | 1.66E-13 | 71/408 |
|  | chromosome | 0005694 | 3.16E-13 | 66/371 |
|  | nucleus | 0005634 | 5.06E-13 | 209/2121 |
|  | chromosome segregation | 0007059 | 9.21E-08 | 33/168 |
|  | cell cycle | 0007049 | 9.21E-08 | 76/608 |
|  | endoplasmic reticulum | 0005783 | 3.38E-07 | 60/448 |
|  | endomembrane system | 0012505 | 5.46E-07 | 70/568 |
|  | signal transduction | 0007165 | 1.65E-06 | 40/259 |
|  | protein modification process | 0006464 | 2.95E-04 | 67/639 |
|  | DNA binding | 0003677 | 3.47E-04 | 61/570 |
|  | DNA metabolic process | 0006259 | 5.90E-04 | 51/460 |
|  | cytoskeleton organization | 0007010 | 5.98E-04 | 30/222 |
|  | protein binding | 0005515 | 6.19E-04 | 69/688 |
|  | transcription | 0006350 | 6.19E-04 | 59/563 |
|  | pseudohyphal growth | 0007124 | 1.16E-03 | 14/73 |
|  | site of polarized growth | 0030427 | 1.47E-03 | 30/237 |
|  | nucleus organization | 0006997 | 2.39E-03 | 13/70 |
|  | conjugation | 0000746 | 2.54E-03 | 18/118 |
|  | ribosome biogenesis | 0042254 | 3.99E-03 | 40/372 |
|  | cellular bud | 0005933 | 1.08E-02 | 24/201 |
|  | cell budding | 0007114 | 1.38E-02 | 13/86 |
|  | helicase activity | 0004386 | 1.40E-02 | 15/107 |
|  | cellular lipid metabolic process | 0044255 | 1.45E-02 | 27/243 |
|  | plasma membrane | 0005886 | 1.46E-02 | 37/366 |
|  | cytoskeleton | 0005856 | 1.50E-02 | 27/245 |
|  | transferase activity | 0016740 | 1.82E-02 | 66/755 |
|  | transcription regulator activity | 0030528 | 1.82E-02 | 36/361 |
|  | protein folding | 0006457 | 1.85E-02 | 15/113 |
|  | RNA metabolic process | 0016070 | 2.46E-02 | 71/837 |
|  | enzyme regulator activity | 0030234 | 2.52E-02 | 26/246 |
|  | signal transducer activity | 0004871 | 3.98E-02 | 10/70 |
|  | nucleolus | 0005730 | 4.32E-02 | 27/271 |

*^a^* Calculated values based on *P* values that calculated by the hypergeometric distribution of one ontology class visualized in the network obtained after FDR was applied.

*^b^* Total number of proteins found in the network which belong to a gene ontology.

*^c^* Total number of proteins that belong to a specific gene ontology.
