# Supplementary material for: Response of Saccharomyces cerevisiae to the Stimulation of Lipopolysaccharide
Source: PLoS One. 2014 Aug 8;9(8):e104428. doi: 10.1371/journal.pone.0104428 (PMC4126697; doi:10.1371/journal.pone.0104428)
Supplement: Table S3 — Selected bottleneck nodes with different gene expression (|Log2R| ≥1.0) observed in networks. (DOC) [file pone.0104428.s003.doc]

**Table S3. Selected bottleneck nodes with different gene expression (|Log_2_R| ≥1.0) observed in networks.**

| Gene/Name | Log_2_R*^d^* | Description |
| --- | --- | --- |
| ***Bottleneck nodes from the induced genes-associated network*** | | |
| YDR171W/HSP42 | 2.87 | Small heat shock protein (sHSP) with chaperone activity; forms barrel-shaped oligomers that suppress unfolded protein aggregation; involved in cytoskeleton reorganization after heat shock |
| YLL026W/HSP104 | 2.26 | Heat shock protein that cooperates with Ydj1p (Hsp40) and Ssa1p (Hsp70) to refold and reactivate previously denatured, aggregated proteins; responsive to stresses including: heat, ethanol, and sodium arsenite; involved in [PSI+] propagation |
| YMR173W/DDR48 | 2.24 | DNA damage-responsive protein, expression is increased in response to heat-shock stress or treatments that produce DNA lesions; contains multiple repeats of the amino acid sequence NNNDSYGS |
| YOR036W/PEP12 | 1.96 | Target membrane receptor (t-SNARE) for vesicular intermediates traveling between the Golgi apparatus and the vacuole; controls entry of biosynthetic, endocytic, and retrograde traffic into the prevacuolar compartment; syntaxin |
| YPL240C/HSP82* | 1.93 | Hsp90 chaperone required for pheromone signaling and negative regulation of Hsf1p; docks with Tom70p for mitochondrial preprotein delivery; promotes telomerase DNA binding and nucleotide addition; interacts with Cns1p, Cpr6p, Cpr7p, Sti1p |
| YNL192W/CHS1 | 1.79 | Chitin synthase I, requires activation from zymogenic form in order to catalyze the transfer of N-acetylglucosamine (GlcNAc) to chitin; required for repairing the chitin septum during cytokinesis; transcription activated by mating factor |
| YHR140W/- | 1.76 | Putative integral membrane protein of unknown function |
| YAR027W/UIP3 | 1.62 | Putative integral membrane protein of unknown function; interacts with Ulp1p at the nuclear periphery; member of DUP240 gene family |
| YJL164C/TPK1* | 1.62 | cAMP-dependent protein kinase catalytic subunit; promotes vegetative growth in response to nutrients via the Ras-cAMP signaling pathway; inhibited by regulatory subunit Bcy1p in the absence of cAMP; partially redundant with Tpk2p and Tpk3p |
| YGL180W/ATG1* | 1.6 | Protein ser/thr kinase required for vesicle formation in autophagy and the cytoplasm-to-vacuole targeting (Cvt) pathway; structurally required for phagophore assembly site formation; during autophagy forms a complex with Atg13p and Atg17p |
| YNR010W/CSE2 | 1.59 | Subunit of the RNA polymerase II mediator complex; associates with core polymerase subunits to form the RNA polymerase II holoenzyme; component of the Med9/10 module; required for regulation of RNA polymerase II activity |
| YJR073C/OPI3* | 1.56 | Phospholipid methyltransferase (methylene-fatty-acyl-phospholipid synthase), catalyzes the last two steps in phosphatidylcholine biosynthesis |
| YBR126C/TPS1 | 1.49 | Synthase subunit of trehalose-6-phosphate synthase/phosphatase complex, which synthesizes the storage carbohydrate trehalose; also found in a monomeric form; expression is induced by the stress response and repressed by the Ras-cAMP pathway |
| YPL026C/SKS1 | 1.36 | Putative serine/threonine protein kinase; involved in the adaptation to low concentrations of glucose independent of the SNF3 regulated pathway |
| YJL130C/URA2 | 1.27 | Bifunctional carbamoylphosphate synthetase (CPSase)-aspartate transcarbamylase (ATCase), catalyzes the first two enzymatic steps in the de novo biosynthesis of pyrimidines; both activities are subject to feedback inhibition by UTP |
| YLR350W/ORM2 | 1.25 | Evolutionarily conserved protein, similar to Orm1p, required for resistance to agents that induce unfolded protein response; Orm1p and Orm2p together control membrane biogenesis by coordinating lipid homeostasis with protein quality control |
| YLL039C/UBI4 | 1.23 | Ubiquitin, becomes conjugated to proteins, marking them for selective degradation via the ubiquitin-26S proteasome system; essential for the cellular stress response; encoded as a polyubiquitin precursor comprised of 5 head-to-tail repeats |
| YJL141C/YAK1 | 1.23 | Serine-threonine protein kinase; component of a glucose-sensing system that inhibits growth in response to glucose availability; upon nutrient deprivation Yak1p phosphorylates Pop2p to regulate mRNA deadenylation, the co-repressor Crf1p to inhibit transcription of ribosomal genes, and the stress-responsive transcription factors Hsf1p and Msn2p; nuclear localization negatively regulated by the Ras/PKA signaling pathway in the presence of glucose |
| YOR209C/NPT1* | 1.22 | Nicotinate phosphoribosyltransferase, acts in the salvage pathway of NAD+ biosynthesis; required for silencing at rDNA and telomeres and has a role in silencing at mating-type loci; localized to the nucleus |
| YAL005C/SSA1 | 1.14 | ATPase involved in protein folding and nuclear localization signal (NLS)-directed nuclear transport; member of heat shock protein 70 (HSP70) family; forms a chaperone complex with Ydj1p; localized to the nucleus, cytoplasm, and cell wall |
| YDL214C/PRR2 | 1.12 | Serine/threonine protein kinase that inhibits pheromone induced signaling downstream of MAPK, possibly at the level of the Ste12p transcription factor; mutant has increased aneuploidy tolerance |
| YNL055C/POR1 | 1.11 | Mitochondrial porin (voltage-dependent anion channel), outer membrane protein required for the maintenance of mitochondrial osmotic stability and mitochondrial membrane permeability; phosphorylated |
| YKL213C/DOA1 | 1.06 | WD repeat protein required for ubiquitin-mediated protein degradation, forms complex with Cdc48p, plays a role in controlling cellular ubiquitin concentration; also promotes efficient NHEJ in postdiauxic/stationary phase |
| YPL057C/SUR1 | 1.05 | Probable catalytic subunit of a mannosylinositol phosphorylceramide (MIPC) synthase, forms a complex with probable regulatory subunit Csg2p; function in sphingolipid biosynthesis is overlapping with that of Csh1p |
| YMR304W/UBP15 | 1.03 | Ubiquitin-specific protease that may play a role in ubiquitin precursor processing |
| YJL165C/HAL5 | 1.00 | Putative protein kinase; overexpression increases sodium and lithium tolerance, whereas gene disruption increases cation and low pH sensitivity and impairs potassium uptake, suggesting a role in regulation of Trk1p and/or Trk2p transporters |
| ***Bottleneck nodes from the repressed genes-associated network*** | | |
| YMR215W/GAS3 | -2.24 | Low abundance, possibly inactive member of the GAS family of GPI-containing proteins; putative 1,3-beta-glucanosyltransferase with similarity to other GAS family members; localizes to the cell wall; mRNA induced during sporulation |
| YCL025C/AGP1 | -1.36 | Low-affinity amino acid permease with broad substrate range, involved in uptake of asparagine, glutamine, and other amino acids; expression is regulated by the SPS plasma membrane amino acid sensor system (Ssy1p-Ptr3p-Ssy5p) |
| YBL002W/HTB2 | -1.34 | Histone H2B, core histone protein required for chromatin assembly and chromosome function; nearly identical to HTB1; Rad6p-Bre1p-Lge1p mediated ubiquitination regulates transcriptional activation, meiotic DSB formation and H3 methylation |
| YNL175C/NOP13 | -1.32 | Nucleolar protein found in preribosomal complexes; contains an RNA recognition motif (RRM) |
| YBL003C/HTA2* | -1.24 | Histone H2A, core histone protein required for chromatin assembly and chromosome function; one of two nearly identical (see also HTA1) subtypes; DNA damage-dependent phosphorylation by Mec1p facilitates DNA repair; acetylated by Nat4p |
| YBR009C/HHF1* | -1.23 | Histone H4, core histone protein required for chromatin assembly and chromosome function; one of two identical histone proteins (see also HHF2); contributes to telomeric silencing; N-terminal domain involved in maintaining genomic integrity |
| YHR169W/DBP8 | -1.22 | ATPase, putative RNA helicase of the DEAD-box family; component of 90S preribosome complex involved in production of 18S rRNA and assembly of 40S small ribosomal subunit; ATPase activity stimulated by association with Esf2p |
| YEL063C/CAN1 | -1.19 | Plasma membrane arginine permease, requires phosphatidyl ethanolamine (PE) for localization, exclusively associated with lipid rafts; mutation confers canavanine resistance |
| YBL039C/URA7 | -1.14 | Major CTP synthase isozyme (see also URA8), catalyzes the ATP-dependent transfer of the amide nitrogen from glutamine to UTP, forming CTP, the final step in *de novo* biosynthesis of pyrimidines; involved in phospholipid biosynthesis |
| YHR052W/CIC1 | -1.13 | Essential protein that interacts with proteasome components and has a potential role in proteasome substrate specificity; also copurifies with 66S pre-ribosomal particles |
| YJR145C/RPS4A | -1.12 | Protein component of the small (40S) ribosomal subunit; mutation affects 20S pre-rRNA processing; identical to Rps4Bp and has similarity to rat S4 ribosomal protein |
| YPL209C/IPL1* | -1.11 | Aurora kinase subunit of the conserved chromosomal passenger complex (CPC; Ipl1p-Sli15p-Bir1p-Nbl1p), involved in regulating kinetochore-microtubule attachments; helps maintain condensed chromosomes during anaphase and early telophase |
| YDR087C/RRP1 | -1.1 | Essential evolutionarily conserved nucleolar protein necessary for biogenesis of 60S ribosomal subunits and processing of pre-rRNAs to mature rRNAs, associated with several distinct 66S pre-ribosomal particles |
| YOR340C/RPA43 | -1.09 | RNA polymerase I subunit A43 |
| YPL211W/NIP7* | -1.07 | Nucleolar protein required for 60S ribosome subunit biogenesis, constituent of 66S pre-ribosomal particles; physically interacts with Nop8p and the exosome subunit Rrp43p |
| YJL098W/SAP185 | -1.07 | Protein that forms a complex with the Sit4p protein phosphatase and is required for its function; member of a family of similar proteins including Sap4p, Sap155p, and Sap190p |
| YCR057C/PWP2 | -1.06 | Conserved 90S pre-ribosomal component essential for proper endonucleolytic cleavage of the 35 S rRNA precursor at A0, A1, and A2 sites; contains eight WD-repeats; PWP2 deletion leads to defects in cell cycle and bud morphogenesis |
| YPL093W/NOG1 | -1.06 | Putative GTPase that associates with free 60S ribosomal subunits in the nucleolus and is required for 60S ribosomal subunit biogenesis; constituent of 66S pre-ribosomal particles; member of the ODN family of nucleolar G-proteins |
| YHR066W/SSF1 | -1.06 | Constituent of 66S pre-ribosomal particles, required for ribosomal large subunit maturation; functionally redundant with Ssf2p; member of the Brix family |
| YBR010W/HHT1 | -1.05 | Histone H3, core histone protein required for chromatin assembly, part of heterochromatin-mediated telomeric and HM silencing; one of two identical histone H3 proteins (see HHT2); regulated by acetylation, methylation, and phosphorylation |
| YPL146C/NOP53 | -1.04 | Nucleolar protein; involved in biogenesis of the 60S subunit of the ribosome; interacts with rRNA processing factors Cbf5p and Nop2p; null mutant is viable but growth is severely impaired |
| YOR210W/RPB10 | -1.04 | RNA polymerase subunit ABC10-beta, common to RNA polymerases I, II, and III |
| YJL033W/HCA4 | -1.03 | Putative nucleolar DEAD box RNA helicase; high-copy number suppression of a U14 snoRNA processing mutant suggests an involvement in 18S rRNA synthesis |
| YDR496C/PUF6 | -1.03 | Pumilio-homology domain protein that binds the 3' UTR of ASH1 mRNA and represses its translation, resulting in proper asymmetric localization of ASH1 mRNA; also co-sediments with the 60S ribosomal subunit and is required for its biogenesis |
| YJR053W/BFA1 | -1.03 | Component of the GTPase-activating Bfa1p-Bub2p complex involved in multiple cell cycle checkpoint pathways that control exit from mitosis |
| YNR053C/NOG2 | -1.02 | Putative GTPase that associates with pre-60S ribosomal subunits in the nucleolus and is required for their nuclear export and maturation |
| YPR119W/CLB2* | -1.01 | B-type cyclin involved in cell cycle progression; activates Cdc28p to promote the transition from G2 to M phase; accumulates during G2 and M, then targeted via a destruction box motif for ubiquitin-mediated degradation by the proteasome |

***** Nodes with high value of betweenness and nodes degree from CenScape.

*^d^* Gene expression difference ratio of Log2 in LPS treated samples compared to control.
