# Supplementary material for: Response of Saccharomyces cerevisiae to the Stimulation of Lipopolysaccharide
Source: PLoS One. 2014 Aug 8;9(8):e104428. doi: 10.1371/journal.pone.0104428 (PMC4126697; doi:10.1371/journal.pone.0104428)
Supplement: Table S4 — Details of genes extracted from Gene Ontology analysis and their classification from the description in SGD. (DOC) [file pone.0104428.s004.doc]

Table S4. Details of genes extracted from Gene Ontology analysis and their classification from the description in SGD.

| Gene/Name | Log_2_R*^d^* | Description |
| --- | --- | --- |
| ***Peroxisome****^e^* |  |  |
| YGL037C/PNC1*^f^* | 1.17 | Nicotinamidase that converts nicotinamide to nicotinic acid as part of the NAD(+) salvage pathway, required for life span extension by calorie restriction; PNC1 expression responds to all known stimuli that extend replicative life span |
| YDL022W/GPD1 | 1.16 | NAD-dependent glycerol-3-phosphate dehydrogenase, key enzyme of glycerol synthesis, essential for growth under osmotic stress; expression regulated by high-osmolarity glycerol response pathway; homolog of Gpd2p |
| YMR304W/UBP15 | 1.03 | Ubiquitin-specific protease that may play a role in ubiquitin precursor processing |
| YGL205W/POX1*^f^* | 2.34 | Fatty-acyl coenzyme A oxidase, involved in the fatty acid beta-oxidation pathway; localized to the peroxisomal matrix |
| YLR284C/ECI1 | 1.51 | Peroxisomal delta3,delta2-enoyl-CoA isomerase, hexameric protein that converts 3-hexenoyl-CoA to trans-2-hexenoyl-CoA, essential for the beta-oxidation of unsaturated fatty acids, oleate-induced |
| YER015W/FAA2 | 1.49 | Medium chain fatty acyl-CoA synthetase, activates imported fatty acids; accepts a wide range of fatty acid chain lengths with a preference for medium chains, C9:0-C13:0; localized to the peroxisome |
| YIR037W/HYR1 | 1.13 | Thiol peroxidase that functions as a hydroperoxide receptor to sense intracellular hydroperoxide levels and transduce a redox signal to the Yap1p transcription factor |
| YGL184C/STR3 | 1.82 | Peroxisomal cystathionine beta-lyase, converts cystathionine into homocysteine; may be redox regulated by Gto1p |
| YKR009C/FOX2 | 1.28 | Multifunctional enzyme of the peroxisomal fatty acid beta-oxidation pathway; has 3-hydroxyacyl-CoA dehydrogenase and enoyl-CoA hydratase activities |
| YJR019C/TES1 | 1.20 | Peroxisomal acyl-CoA thioesterase likely to be involved in fatty acid oxidation rather than fatty acid synthesis; conserved protein also found in human peroxisomes; TES1 mRNA levels increase during growth on fatty acids |
| YKL188C/PXA2 | 1.47 | Subunit of a heterodimeric peroxisomal ATP-binding cassette transporter complex (Pxa1p-Pxa2p), required for import of long-chain fatty acids into peroxisomes; similarity to human adrenoleukodystrophy transporter and ALD-related proteins |
| YHR160C/PEX18*^f^* | 2.19 | Peroxin required for targeting of peroxisomal matrix proteins containing PTS2; interacts with Pex7p; partially redundant with Pex21p |
| YIL160C/POT1*^f^* | 1.58 | 3-ketoacyl-CoA thiolase with broad chain length specificity, cleaves 3-ketoacyl-CoA into acyl-CoA and acetyl-CoA during beta-oxidation of fatty acids |
| YNL009W/IDP3 | 1.27 | Peroxisomal NADP-dependent isocitrate dehydrogenase, catalyzes oxidation of isocitrate to alpha-ketoglutarate with the formation of NADP(H+), required for growth on unsaturated fatty acids |
| YML042W/CAT2 | 1.18 | Carnitine acetyl-CoA transferase present in both mitochondria and peroxisomes, transfers activated acetyl groups to carnitine to form acetylcarnitine which can be shuttled across membranes |
| ***Mitochondrial inner membrane****^e^* | | |
| YAL039C/CYC3 | 1.35 | Cytochrome c heme lyase (holocytochrome c synthase), attaches heme to apo-cytochrome c (Cyc1p or Cyc7p) in the mitochondrial intermembrane space; human ortholog may have a role in microphthalmia with linear skin defects (MLS) |
| YJL166W/QCR8 | 1.16 | Subunit 8 of ubiquinol cytochrome-c reductase complex, which is a component of the mitochondrial inner membrane electron transport chain; oriented facing the intermembrane space; expression is regulated by Abf1p and Cpf1p |
| YML120C/NDI1*^f^* | 1.39 | NADH:ubiquinone oxidoreductase, transfers electrons from NADH to ubiquinone in the respiratory chain but does not pump protons, in contrast to the higher eukaryotic multisubunit respiratory complex I; phosphorylated; homolog of human AMID |
| YIL155C/GUT2 | 1.30 | Mitochondrial glycerol-3-phosphate dehydrogenase; expression is repressed by both glucose and cAMP and derepressed by non-fermentable carbon sources in a Snf1p, Rsf1p, Hap2/3/4/5 complex dependent manner |
| YGR243W/FMP43 | 1.39 | Putative protein of unknown function; expression regulated by osmotic and alkaline stresses; the authentic, non-tagged protein is detected in highly purified mitochondria in high-throughput studies |
| YPR191W/QCR2 | 1.03 | Subunit 2 of the ubiquinol cytochrome-c reductase complex, which is a component of the mitochondrial inner membrane electron transport chain; phosphorylated; transcription is regulated by Hap1p, Hap2p/Hap3p, and heme |
| YLR348C/DIC1 | 1.12 | Mitochondrial dicarboxylate carrier, integral membrane protein, catalyzes a dicarboxylate-phosphate exchange across the inner mitochondrial membrane, transports cytoplasmic dicarboxylates into the mitochondrial matrix |
| YDR178W/SDH4 | 1.16 | Membrane anchor subunit of succinate dehydrogenase (Sdh1p, Sdh2p, Sdh3p, Sdh4p), which couples the oxidation of succinate to the transfer of electrons to ubiquinone as part of the TCA cycle and the mitochondrial respiratory chain |
| YLL041C/SDH2 | 1.09 | Iron-sulfur protein subunit of succinate dehydrogenase (Sdh1p, Sdh2p, Sdh3p, Sdh4p), which couples the oxidation of succinate to the transfer of electrons to ubiquinone as part of the TCA cycle and the mitochondrial respiratory chain |
| YEL024W/RIP1 | 1.22 | Ubiquinol-cytochrome-c reductase, a Rieske iron-sulfur protein of the mitochondrial cytochrome bc1 complex; transfers electrons from ubiquinol to cytochrome c1 during respiration |
| YPL134C/ODC1 | 1.22 | Mitochondrial inner membrane transporter, exports 2-oxoadipate and 2-oxoglutarate from the mitochondrial matrix to the cytosol for lysine and glutamate biosynthesis and lysine catabolism; suppresses, in multicopy, an fmc1 null mutation |
| YMR035W/IMP2 | 1.36 | Catalytic subunit of the mitochondrial inner membrane peptidase complex, required for maturation of mitochondrial proteins of the intermembrane space; complex contains Imp1p and Imp2p (both catalytic subunits), and Som1p |
| YEL059C-A/SOM1 | 1.08 | Subunit of the mitochondrial inner membrane peptidase, which is required for maturation of mitochondrial proteins of the intermembrane space; Som1p facilitates cleavage of a subset of substrates; contains twin cysteine-x9-cysteine motifs |
| YML042W/CAT2 | 1.18 | Carnitine acetyl-CoA transferase present in both mitochondria and peroxisomes, transfers activated acetyl groups to carnitine to form acetylcarnitine which can be shuttled across membranes |
| ***Mitochondrial intermembrane space****^e^* | | |
| YBR056W/- | 1.97 | Putative cytoplasmic protein of unknown function |
| YKL067W/YNK1 | 1.33 | Nucleoside diphosphate kinase, catalyzes the transfer of gamma phosphates from nucleoside triphosphates, usually ATP, to nucleoside diphosphates by a mechanism that involves formation of an autophosphorylated enzyme intermediate |
| YAL039C/CYC3 | 1.35 | Cytochrome c heme lyase (holocytochrome c synthase), attaches heme to apo-cytochrome c (Cyc1p or Cyc7p) in the mitochondrial intermembrane space; human ortholog may have a role in microphthalmia with linear skin defects (MLS) |
| YKL150W/MCR1 | 1.07 | Mitochondrial NADH-cytochrome b5 reductase, involved in ergosterol biosynthesis |
| YIR037W/HYR1 | 1.13 | Thiol peroxidase that functions as a hydroperoxide receptor to sense intracellular hydroperoxide levels and transduce a redox signal to the Yap1p transcription factor |
| YDL085W/NDE2 | 1.78 | Mitochondrial external NADH dehydrogenase, catalyzes the oxidation of cytosolic NADH; Nde1p and Nde2p are involved in providing the cytosolic NADH to the mitochondrial respiratory chain |
| YJL066C/MPM1 | 1.14 | Mitochondrial membrane protein of unknown function, contains no hydrophobic stretches |
| YIL160C/POT1 | 1.58 | 3-ketoacyl-CoA thiolase with broad chain length specificity, cleaves 3-ketoacyl-CoA into acyl-CoA and acetyl-CoA during beta-oxidation of fatty acids |
| ***Mitochondrial outer membrane****^e^* | | |
| YPL186C/UIP4 | 2.50 | Protein that interacts with Ulp1p, a Ubl (ubiquitin-like protein)-specific protease for Smt3p protein conjugates; detected in a phosphorylated state in the mitochondrial outer membrane; also detected in ER and nuclear envelope |
| YIR038C/GTT1 | 1.95 | ER associated glutathione S-transferase capable of homodimerization; expression induced during the diauxic shift and throughout stationary phase; functional overlap with Gtt2p, Grx1p, and Grx2p |
| YIL136W/OM45 | 1.86 | Protein of unknown function, major constituent of the mitochondrial outer membrane; located on the outer (cytosolic) face of the outer membrane |
| YOR285W/RDL1 | 1.73 | Protein of unknown function containing a rhodanese-like domain; localized to the mitochondrial outer membrane |
| YNL055C/POR1*^f^* | 1.11 | Mitochondrial porin (voltage-dependent anion channel), outer membrane protein required for the maintenance of mitochondrial osmotic stability and mitochondrial membrane permeability; phosphorylated |
| YKL150W/MCR1 | 1.07 | Mitochondrial NADH-cytochrome b5 reductase, involved in ergosterol biosynthesis |
| ***Protein folding****^e^* | | |
| YBR072W/HSP26*^f^* | 4.24 | Small heat shock protein (sHSP) with chaperone activity; forms hollow, sphere-shaped oligomers that suppress unfolded proteins aggregation; oligomer activation requires heat-induced conformational change; also has mRNA binding activity |
| YPL240C/HSP82 | 1.93 | Hsp90 chaperone required for pheromone signaling and negative regulation of Hsf1p; docks with Tom70p for mitochondrial preprotein delivery; promotes telomerase DNA binding and nucleotide addition; interacts with Cns1p, Cpr6p, Cpr7p, Sti1p |
| YBR169C/SSE2 | 1.79 | Member of the heat shock protein 70 (HSP70) family; may be involved in protein folding; localized to the cytoplasm; highly homologous to the heat shock protein Sse1p |
| YER103W/SSA4 | 1.72 | Heat shock protein that is highly induced upon stress; plays a role in SRP-dependent cotranslational protein-membrane targeting and translocation; member of the HSP70 family; cytoplasmic protein that concentrates in nuclei upon starvation |
| YNL007C/SIS1 | 1.31 | Type II HSP40 co-chaperone that interacts with the HSP70 protein Ssa1p; not functionally redundant with Ydj1p due to due to substrate specificity; shares similarity with bacterial DnaJ proteins |
| YAL005C/SSA1 | 1.14 | ATPase involved in protein folding and nuclear localization signal (NLS)-directed nuclear transport; member of heat shock protein 70 (HSP70) family; forms a chaperone complex with Ydj1p; localized to the nucleus, cytoplasm, and cell wall |
| YBR208C/DUR1,2 | -1.08 | Urea amidolyase, contains both urea carboxylase and allophanate hydrolase activities, degrades urea to CO2 and NH3; expression sensitive to nitrogen catabolite repression and induced by allophanate, an intermediate in allantoin degradation |
| YHR176W/FMO1 | 1.64 | Flavin-containing monooxygenase, localized to the cytoplasmic face of the ER membrane; catalyzes oxidation of biological thiols to maintain the ER redox buffer ratio for correct folding of disulfide-bonded proteins |
| YGR142W/BTN2 | 1.65 | v-SNARE binding protein that facilitates specific protein retrieval from a late endosome to the Golgi; modulates arginine uptake, possible role in mediating pH homeostasis between the vacuole and plasma membrane H(+)-ATPase |
| YPR158W/CUR1 | 1.23 | Protein of unknown function involved in destabilization of |
| ***Autophagy****^e^* | | |
| YLR356W/ATG33 | 1.52 | Mitochondrial mitophagy-specific protein; required primarily for mitophagy induced at the post-log phase; not required for other types of selective autophagy or macroautophagy; conserved within fungi, but not in higher eukaryotes |
| YOL082W/ATG19 | 1.54 | Receptor protein specific for the cytoplasm-to-vacuole targeting (Cvt) pathway; delivers cargo proteins aminopeptidase I (Lap4p) and alpha-mannosidase (Ams1p) to the phagophore assembly site for packaging into Cvt vesicles |
| YGL180W/ATG1*^f^* | 1.60 | Protein ser/thr kinase required for vesicle formation in autophagy and the cytoplasm-to-vacuole targeting (Cvt) pathway; structurally required for phagophore assembly site formation; during autophagy forms a complex with Atg13p and Atg17p |
| YBL078C/ATG8*^f^* | 1.41 | Component of autophagosomes and Cvt vesicles; undergoes conjugation to phosphatidylethanolamine (PE); Atg8p-PE is anchored to membranes, is involved in phagophore expansion, and may mediate membrane fusion during autophagosome formation |
| YPL149W/ATG5 | 1.40 | Conserved protein involved in autophagy and the Cvt pathway; undergoes conjugation with Atg12p to form a complex involved in Atg8p lipidation; conjugated Atg12p also forms a complex with Atg16p that is essential for autophagosome formation |
| YOL083W/ATG34 | 1.44 | Receptor protein involved in selective autophagy during starvation; specifically involved in the transport of cargo protein alpha-mannosidase (Ams1p); Atg19p paralog |
| YDL113C/ATG20 | 1.07 | Sorting nexin family member required for the cytoplasm-to-vacuole targeting (Cvt) pathway and for endosomal sorting; has a Phox homology domain that binds phosphatidylinositol-3-phosphate; interacts with Snx4p; potential Cdc28p substrate |
| YDL149W/ATG9 | 1.16 | Transmembrane protein involved in forming Cvt and autophagic vesicles; cycles between the phagophore assembly site (PAS) and other cytosolic punctate structures, not found in autophagosomes; may be involved in membrane delivery to the PAS |
| YCR068W/ATG15*^f^* | 1.16 | Lipase required for intravacuolar lysis of autophagic bodies and Cvt bodies; targeted to intravacuolar vesicles during autophagy via the multivesicular body (MVB) pathway |
| YPL166W/ATG29 | 1.03 | Autophagy-specific protein that is required for recruitment of other ATG proteins to the pre-autophagosomal structure (PAS); interacts with Atg17p and localizas to the PAS in a manner interdependent with Atg17p and Cis1p; not conserved |
| ***Endocytosis****^e^* | | |
| YBR214W/SDS24 | 1.70 | One of two *S. cerevisiae* homologs (Sds23p and Sds24p) of the S. pombe Sds23 protein, which is implicated in APC/cyclosome regulation; involved in cell separation during budding; may play an indirect role in fluid-phase endocytosis |
| YKL129C/MYO3 | 1.27 | One of two type I myosins; localizes to actin cortical patches; deletion of MYO3 has little effect on growth, but myo3 myo5 double deletion causes severe defects in growth and actin cytoskeleton organization |
| YML052W/SUR7 | -1.34 | Plasma membrane protein that localizes to furrow-like invaginations (MCC patches); component of eisosomes; associated with endocytosis, along with Pil1p and Lsp1p; sporulation and plasma membrane sphingolipid content are altered in mutants |
| YNL093W/YPT53*^f^* | 3.67 | Rab family GTPase, similar to Ypt51p and Ypt52p and to mammalian rab5; required for vacuolar protein sorting and endocytosis |
| YPR030W/CSR2*^f^* | 2.98 | GTPase-activating protein for Gpa1p; regulates endocytosis |
| YOR237W/HES1 | 1.89 | Protein implicated in the regulation of ergosterol biosynthesis; one of a seven member gene family with a common essential function and non-essential unique functions; similar to human oxysterol binding protein (OSBP) |
| YNL055C/POR1 | 1.11 | Mitochondrial porin (voltage-dependent anion channel), outer membrane protein required for the maintenance of mitochondrial osmotic stability and mitochondrial membrane permeability; phosphorylated |
| ***Apoptosis****^e^* | | |
| YPL123C/RNY1*^f^* | 1.84 | Vacuolar RNase of the T(2) family, relocalizes to the cytosol where it cleaves tRNAs upon oxidative or stationary phase stress; promotes apoptosis under stress conditions and this function is independent of its catalytic activity |
| YNL305C/BXI1*^f^* | 1.40 | Protein involved in apoptosis; variously described as containing a BCL-2 homology (BH3) domain or as a member of the BAX inhibitor family; reported to promote apoptosis under some conditions and to inhibit it in others; localizes to ER and vacuole; may link the unfolded protein response to apoptosis via regulation of calcium-mediated signaling; translocates to mitochondria under apoptosis-inducing conditions in a process involving Mir1p and Cor1p |
| YPL171C/OYE3 | 2.17 | Conserved NADPH oxidoreductase containing flavin mononucleotide (FMN), homologous to Oye2p with different ligand binding and catalytic properties; has potential roles in oxidative stress response and programmed cell death |
| ***Ribosome biogenesis****^e^* | | |
| YNL112W/DBP2 | -1.39 | Essential ATP-dependent RNA helicase of the DEAD-box protein family, involved in nonsense-mediated mRNA decay and rRNA processing |
| YOR145C/PNO1 | -1.06 | Essential nucleolar protein required for pre-18S rRNA processing, interacts with Dim1p, an 18S rRNA dimethyltransferase, and also with Nob1p, which is involved in proteasome biogenesis; contains a KH domain |
| YPL093W/NOG1 | -1.06 | Putative GTPase that associates with free 60S ribosomal subunits in the nucleolus and is required for 60S ribosomal subunit biogenesis; constituent of 66S pre-ribosomal particles; member of the ODN family of nucleolar G-proteins |
| YMR131C/RRB1 | -1.05 | Essential nuclear protein involved in early steps of ribosome biogenesis; physically interacts with the ribosomal protein Rpl3p |
| YNR053C/NOG2 | -1.02 | Putative GTPase that associates with pre-60S ribosomal subunits in the nucleolus and is required for their nuclear export and maturation |
| YCR057C/PWP2 | -1.06 | Conserved 90S pre-ribosomal component essential for proper endonucleolytic cleavage of the 35 S rRNA precursor at A0, A1, and A2 sites; contains eight WD-repeats; PWP2 deletion leads to defects in cell cycle and bud morphogenesis |
| YJL033W/HCA4 | -1.03 | Putative nucleolar DEAD box RNA helicase; high-copy number suppression of a U14 snoRNA processing mutant suggests an involvement in 18S rRNA synthesis |
| YHR087W/RTC3 | 2.09 | Protein of unknown function involved in RNA metabolism; has structural similarity to SBDS, the human protein mutated in Shwachman-Diamond Syndrome (the yeast SBDS ortholog = SDO1); null mutation suppresses cdc13-1 temperature sensitivity |
| YHR197W/RIX1 | -1.20 | Essential component of the Rix1 complex (Rix1p, Ipi1p, Ipi3p) that is required for processing of ITS2 sequences from 35S pre-rRNA; Rix1 complex associates with Mdn1p in pre-60S ribosomal particles |
| YCR072C/RSA4 | -1.10 | WD-repeat protein involved in ribosome biogenesis; may interact with ribosomes; required for maturation and efficient intra-nuclear transport or pre-60S ribosomal subunits, localizes to the nucleolus |
| YLL011W/SOF1 | -1.15 | Essential protein required for biogenesis of 40S (small) ribosomal subunit; has similarity to the beta subunit of trimeric G-proteins and the splicing factor Prp4p |
| YKR024C/DBP7 | -1.09 | Putative ATP-dependent RNA helicase of the DEAD-box family involved in ribosomal biogenesis; essential for growth under anaerobic conditions |
| YHR066W/SSF1 | -1.06 | Constituent of 66S pre-ribosomal particles, required for ribosomal large subunit maturation; functionally redundant with Ssf2p; member of the Brix family |
| YHR052W/CIC1 | -1.13 | Essential protein that interacts with proteasome components and has a potential role in proteasome substrate specificity; also copurifies with 66S pre-ribosomal particles |
| YOR004W/UTP23 | -1.23 | Essential nucleolar protein that is a component of the SSU (small subunit) processome involved in 40S ribosomal subunit biogenesis; has homology to PINc domain protein Fcf1p, although the PINc domain of Utp23p is not required for function |
| YPL146C/NOP53 | -1.04 | Nucleolar protein; involved in biogenesis of the 60S subunit of the ribosome; interacts with rRNA processing factors Cbf5p and Nop2p; null mutant is viable but growth is severely impaired |
| YAL059W/ECM1 | -1.24 | Pre-ribosomal factor involved in 60S ribosomal protein subunit export; associates with the pre-60S particle; shuttles between the nucleus and cytoplasm |
| YIL019W/FAF1 | -1.50 | Protein required for pre-rRNA processing and 40S ribosomal subunit assembly |
| YPL081W/RPS9A | -1.03 | Protein component of the small (40S) ribosomal subunit; nearly identical to Rps9Bp and has similarity to *E. coli* S4 and rat S9 ribosomal proteins |
| YDR449C/UTP6 | -1.04 | Nucleolar protein, component of the small subunit (SSU) processome containing the U3 snoRNA that is involved in processing of pre-18S rRNA |
| YHR169W/DBP8 | -1.22 | ATPase, putative RNA helicase of the DEAD-box family; component of 90S preribosome complex involved in production of 18S rRNA and assembly of 40S small ribosomal subunit; ATPase activity stimulated by association with Esf2p |
| YNL182C/IPI3 | -1.08 | Essential component of the Rix1 complex (Rix1p, Ipi1p, Ipi3p) that is required for processing of ITS2 sequences from 35S pre-rRNA; highly conserved and contains WD40 motifs; Rix1 complex associates with Mdn1p in pre-60S ribosomal particles |
| YHR085W/IPI1 | -1.15 | Essential component of the Rix1 complex (with Rix1p and Ipi3p) that is required for processing of ITS2 sequences from 35S pre-rRNA; Rix1 complex associates with Mdn1p in pre-60S ribosomal particles |
| YGL171W/ROK1 | -1.00 | ATP-dependent RNA helicase of the DEAD box family; required for 18S rRNA synthesis |
| YDR021W/FAL1 | -1.20 | Nucleolar protein required for maturation of 18S rRNA, member of the eIF4A subfamily of DEAD-box ATP-dependent RNA helicases |
| YKL078W/DHR2 | -1.41 | Predominantly nucleolar DEAH-box ATP-dependent RNA helicase, required for 18S rRNA synthesis |
| YKR060W/UTP30 | -1.03 | Subunit of U3-containing 90S preribosome complex involved in production of 18S rRNA and assembly of small ribosomal subunit |
| YPL211W/NIP7 | -1.07 | Nucleolar protein required for 60S ribosome subunit biogenesis, constituent of 66S pre-ribosomal particles; physically interacts with Nop8p and the exosome subunit Rrp43p |
| ***ATP deficient upon negatively regulation*** | | |
| YDL130W-A/STF1 | 2.35 | Regulation of the mitochondrial F1F0-ATP synthase |
| YJL116C/NCA3 | 2.01 | Mitochondrion organization; with Nca2p to regulate mitochondrial expression of subunits 6 (Atp6p) and 8 (Atp8p) of the F0-F1 ATP |
| YDL181W/INH1 | 1.36 | Inhibits ATP hydrolysis by the F1F0-ATP synthase |
| ***Targeting to vacuolar*** | | |
| YEL060C/PRB1*^f^* | 2.90 | Vacuolar proteinase B; involved in breakdown of intravacuolar vesicles |
| YGL156W/AMS1*^f^* | 2.57 | Vacuolar alpha mannosidase; involved in free oligosaccharide (fOS) degradation |
| YMR040W/YET2 | 2.44 | Homolog of human BAP31 protein; may role in transport across ER |
| YNL015W/PBI2 | 2.28 | Assists in priming SNARE molecules and promotes vacuole fusion |
| YDL234C/GYP7 | 2.27 | GTPase-activating protein for yeast Rab family members; involved in vesicle mediated protein trafficking |
| YBR005W/RCR1 | 2.05 | Protein of the ER membrane involved in cell wall chitin deposition; endosomal-vacuolar trafficking pathway |
| YOR036W/PEP12 | 1.96 | Target membrane receptor; intermediates traveling between the Golgi apparatus and the vacuole |
| YLR080W/EMP46 | 1.64 | Integral membrane component of ER-derived COPII-coated vesicles; functions in ER to Golgi transport |
| YIL017C/VID28 | 1.48 | GID Complex subunit, adaptor for regulatory subunit Vid24p;involved in proteasome-dependent catabolite degradation of FBPase |
| YGL227W/VID30 | 1.30 | Required for association of Vid vesicles and actin patches in vacuole import and degradation pathway; involved in FBPase degradation |
| YKL103C/APE1*^f^* | 1.25 | Vacuolar aminopeptidase yscI; a marker protein in studies of autophagy and cytosol to vacuole targeting (CVT) pathway |
| YJL151C/SNA3 | 1.23 | Involved in efficient MVB sorting of proteins to the vacuole; may function as an RSP5 adapter protein for MVB cargos |
| YMR196W/- | 1.15 | (GFP)-fusion protein localizes to the cytoplasm |
| YOL129W/VPS68 | 1.06 | Vacuolar membrane protein; involved in vacuolar protein sorting |
| ***Chaperones mediated*** | | |
| YDR171W/HSP42 | 2.87 | Similar to HSP26; involved in cytoskeleton reorganization; functions in both unstressed and stressed cells |
| YLL026W/HSP104 | 2.26 | Disaggregase; heat shock protein that cooperates with Ydj1p (Hsp40) and Ssa1p (Hsp70) to refold and reactivate previously denatured, aggregated proteins |
| YDR258C/HSP78 | 1.62 | Oligomeric mitochondrial matrix chaperone; with Ssc1p in mitochondrial thermotolerance after heat shock; prevent the aggregation of misfolded proteins and resolubilize protein aggregates |
| ***Ubiquitin mediated*** | | |
| YEL012W/UBC8 | 1.56 | Mediates the glucose-induced ubiquitination of FBPase; catalyzes the ubiquitination of histones |
| YJL048C/UBX6 | 1.52 | UBX (ubiquitin regulatory X) domain-containing protein that interacts with Cdc48p |
| YNR069C/BSC5 | 1.33 | Involved in ubiquitin-mediated sorting of plasma membrane proteins |
| YLL039C/UBI4 | 1.23 | Conjugated to proteins for selective degradation via the ubiquitin-26S proteasome system; for the cellular stress response; as a polyubiquitin precursor comprised of 5 head-to-tail repeats |
| YER144C/UBP5 | 1.23 | Putative ubiquitin-specific protease;concentrates at the bud neck |
| YDR059C/UBC5 | 1.22 | Ubiquitin-conjugating enzyme that mediates selective degradation of short-lived, abnormal, or excess proteins, including histone H3; central component of the cellular stress response |
| YJR036C/HUL4 | 1.16 | Similarity to hect domain E3 ubiquitin-protein ligases |
| YJL149W/DAS1 | 1.09 | Putative SCF ubiquitin ligase F-box protein; similar to putative F-box protein YDR131C |
| YKL213C/DOA1 | 1.06 | WD repeat protein required for ubiquitin-mediated protein degradation; role in controlling cellular ubiquitin concentration |
| YNR068C/BUL3 | 1.02 | Expressed as a readthrough product of BSC5, the readthrough locus being termed BUL3; the BUL3 readthrough product is involved in ubiquitin-mediated sorting of plasma membrane proteins |
| YOR257W/CDC31 | 1.01 | Binds multiubiquitinated proteins and involved in proteasomal protein degradation |
| YLR102C/APC9 | 1.00 | Subunit of the APC/C, a ubiquitin-protein ligase required for degradation of anaphase inhibitors during the metaphase/anaphase transition |
| ***ROS prevention*** | | |
| YGR256W/GND2 | 3.55 | Catalyzes the second oxidative reduction of NADP^+^ to NADPH; protects yeast from oxidative stress |
| YLR327C/TMA10 | 3.49 | Unknown function that associates with ribosomes; paralog of STF2 |
| YGR088W/CTT1 | 2.47 | Cytosolic catalase T, has a role in protection from oxidative damage by hydrogen peroxide |
| YDR453C/TSA2 | 2.35 | Cooperates with Tsa1p in the removal of reactive oxygen |
| YGR008C/STF2*^f^* | 1.66 | Antioxidant, overexpression prevents ROS accumulation and apoptosis |
| YCL035C/GRX1 | 1.61 | Protects cells from oxidative damage |
| YKL026C/GPX1 | 1.49 | Protects cells from phospholipid hydroperoxides and nonphospholipid peroxides during oxidative stress |
| YBL064C/PRX1 | -1.25 | Pre-ribosomal factor involved in 60S ribosomal protein subunit export; associates with the pre-60S particle |
| YNR064C/- | 1.34 | Alpha/beta hydrolase fold family; role in detoxification of epoxides |
| YHR008C/SOD2*^f^* | 1.09 | Mitochondrial manganese superoxide dismutase, protects cells against oxygen toxicity; |
| YBR244W/GPX2 | 1.06 | Protects cells from phospholipid hydroperoxides and nonphospholipid peroxides during oxidative stress |

*^d^* Gene expression difference ratio of Log2 in LPS treated samples compared to control.

*^e^* Gene Ontology categories with the corresponding genes mentioned in the manuscript.

*^f^* Genes picked up for single gene knockout mutant analysis.
