# Supplementary material for: Response of Saccharomyces cerevisiae to the Stimulation of Lipopolysaccharide
Source: PLoS One. 2014 Aug 8;9(8):e104428. doi: 10.1371/journal.pone.0104428 (PMC4126697; doi:10.1371/journal.pone.0104428)
Supplement: Table S5 — Other Genes with the expression ratio of Log2 more than 2 or less than -0.5 found in LPS-treated S. cerevisiae BY4742 cells. (DOC) [file pone.0104428.s005.doc]

**Table S5. Other Genes with the expression ratio of Log2 more than 2 or less than -0.5 found in LPS-treated *S. cerevisiae*** BY4742 cells.

| Gene/Name | Log_2_R*^d^* | Description |
| --- | --- | --- |
| ***Cell wall stress*** |  |  |
| YOR134W/BAG7 | 3.00 | Rho GTPase activating protein;stimulates the intrinsic GTPase activity in regulating actin cytoskeleton organization and cell wall biosynthesis |
| YOR382W/FIT2 | 2.63 | Mannoprotein that is incorporated into the cell wall via a GPI anchor; involved in the retention of siderophore-iron in the cell wall |
| YLR121C/YPS3 | 2.24 | Member of the yapsin family of proteases involved in cell wall growth and maintenance; attached to the plasma membrane via a GPI anchor |
| YDR055W/PST1 | 2.12 | Cell wall protein with putative GPI-attachment site; up-regulated by activation of the cell integrity pathway |
| YLR120C/YPS1 | 2.04 | Member of the yapsin family of proteases involved in cell wall growth and maintenance; attached to the plasma membrane via a GPI anchor |
| YHR139C/SPS100 | 1.81 | Protein required for spore wall maturation; induced in cells treated with the mycotoxin patulin |
| YKL096W/CWP1 | -3.47 | Cell wall mannoprotein that localizes to birth scars of daughter cells; linked to a beta-1,3- and beta-1,6-glucan heteropolymer through a phosphodiester bond; required for propionic acid resistance |
| YER011W/TIR1 | -2.64 | Cell wall mannoprotein of the Srp1p/Tip1p family; downregulated at acidic pH |
| YEL040W/UTR2 | -2.56 | Chitin transglycosylase for glucans biogenesis; (GPI)-anchored protein localized to bud neck |
| YMR215W/GAS3 | -2.25 | Putative 1,3-beta-glucanosyltransferase; possibly inactive member of the GAS family of GPI-containing proteins; localizes to the cell wall |
| ***Other stress induced*** | | |
| YOR391C/HSP33 | 3.19 | Possible chaperone and cysteine protease; member of DJ-1/ThiJ/PfpI superfamily |
| YMR107W/SPG4 | 3.18 | Protein required for high temperature survival during stationary phase |
| YOL052C-A/DDR2 | 2.92 | Multi-stress response protein; expression is activated by a variety of xenobiotic agents and environmental or physiological stresses |
| YDR533C/HSP31 | 2.65 | Methylglyoxalase that converts methylglyoxal to D-lactate; member of DJ-1/ThiJ/PfpI superfamily; involved in oxidative stress resistance and expression is induced under oxidative stress; |
| YMR169C/ALD3 | 2.65 | Cytoplasmic aldehyde dehydrogenase; involved in beta-alanine synthesis; expression is induced by stress and repressed by glucose |
| YOR220W/RCN2 | 2.43 | Phosphorylated in response to alpha factor |
| YBR117C/TKL2 | 2.40 | Induced in carbon-limited (low glucose) cultures, in response to lithium chloride or dimethyl sulfoxide (DMSO), and at the diauxic transition in a Msn2/4p-dependent manner |
| YBR203W/COS111 | 2.34 | Resistance to the antifungal drug ciclopirox olamine; not related to COS family |
| YMR173W/DDR48 | 2.24 | Increased in response to heat-shock stress or treatments that produce DNA lesions |
| YGR052W/FMP48 | 2.02 | Induced by treatment with 8-methoxypsoralen and UVA irradiation |
| YPL280W/HSP32 | 1.83 | Possible chaperone and cysteine protease; member of DJ-1/ThiJ/PfpI superfamily |
| ***Ion homeostasis*** | | |
| YLL052C/AQY2 | 3.43 | Water channel across cell membranes ; controlled by osmotic signals; involved in freeze tolerance |
| YJL144W/YJL144W | 3.16 | Cytoplasmic hydrophilin essential in dessication-rehydration process ; induced by osmotic stress, starvation and during stationary phase |
| YOL016C/CMK2 | 2.66 | Calmodulin-dependent protein kinase; role in stress response, many Ca^2+^/calmodulan dependent phosphorylation substrates demonstrated *in vitro*,amino acid sequence similar to mammalian Cam Kinase II |
| YGR032W/GSC2 | 2.57 | Catalytic subunit; formation of the inner layer of the spore wall; ositively regulated by Rho1p; expression induced by calcium |
| YOL084W/PHM7 | 2.49 | Regulated by phosphate levels; cell periphery and vacuole |
| YLR136C/TIS11 | 2.31 | mRNA-binding protein expressed during iron starvation; involved in iron homeostasis |
| YBR296C/PHO89 | 2.19 | Na^+^/Pi cotransporter; regulated by inorganic phosphate concentrations and Pho4p |
| YOL152W/FRE7 | 2.18 | Putative ferric reductase; induced by low copper levels |
| YHL040C/ARN1 | 2.11 | ARN family transporter; uptake of iron |
| ***Cell fusion*** | | |
| YBR040W/FIG1 | -3.18 | Integral membrane protein; low affinity Ca^2+^ influx system related about intracellular signaling and cell-cell fusion |
| YJR004C/SAG1 | -2.47 | Alpha-agglutinin of alpha-cells; binds to Aga1p during agglutination; N-terminal half: homologous to the immunoglobulin superfamily; C-terminal half: highly glycosylated and contains GPI anchor |
| YML047C/PRM6 | -2.40 | Localized to sites of polarized growth; up-regulated in response to alpha factor |
| YGL089C/MF(ALPHA)2 | -2.32 | Mating pheromone alpha-factor, made by alpha cells; also encoded by MF(ALPHA)1, which is more highly expressed |
| YNR044W/AGA1 | -2.18 | Anchorage subunit of a-agglutinin;highly O-glycosylated protein with N-terminal;C-terminal signal for addition of GPI anchor to cell wall |
| YDL227C/HO | -2.06 | Site-specific endonuclease; required for gene conversion at the MAT locus (homothallic switching) through the generation of a ds DNA break |
| ***Unknown*** | | |
| YPR145C-A/- | 4.27 | Unknown |
| YPL278C/- | 4.19 | Unknown function; gene expression regulated by copper levels |
| YDR034W-B/- | 3.67 | Unknown tail-anchored plasma membrane protein; conserved CYSTM module |
| YPL054W/LEE1 | 3.57 | Unknown |
| YLL053C/- | 3.49 | Unknown protein contiguous with AQY2 which encodes an aquaporin |
| YPL277C/- | 3.40 | Unknown function localized to the membranes; gene expression regulated by copper levels |
| YOR385W/- | 3.09 | Unknown protein in cytoplasm |
| YAL064W-B/- | 2.90 | Fungal-specific protein of unknown function |
| YNL092W/- | 2.88 | Putative S-adenosylmethionine-dependent methyltransferase of the seven beta-strand family |
| YHR138C/- | 2.84 | Unknown protein; similar to Pbi2p; double null mutant lacking Pbi2p and Yhr138cp exhibits highly fragmented vacuoles |
| YLR149C/- | 2.75 | Unknown protein; overexpression causes a cell cycle delay or arrest; null mutation results in a decrease in plasma membrane electron transport |
| YMR084W/- | 2.61 | Unknown |
| YNL208W/- | 2.61 | Unknown |
| YNR034W-A/- | 2.46 | Unknown function regulated by Msn2p/Msn4p |
| YOR389W/- | 2.45 | Unknown function regulated by copper levels |
| YGR248W/- | 2.43 | 6-phosphogluconolactonase;DNA replication stress |
| YCR007C/- | 2.40 | Putative integral membrane protein, DUP240 gene family; |
| YMR085W/- | 2.24 | paralogous to glutamine-fructose-6-phosphate amidotransferase GFA1 |
| YMR034C/- | 2.23 | Putative transporter;SLC10 carrier family; involved in azole resistance |
| YBR085C-A/- | 2.05 | Unknown function; protein abundance increases in response to DNA replication stress |
| YJR061W/MNN14 | 2.02 | Unknown function; transcription repressed by Rm101p |
| YMR230W-A/- | -2.56 | Unknown |
| YOR214C/- | -2.12 | Unknown function; paralogous to SPO19 |
| YGL101W/- | -2.00 | Unknown protein; interacts with the DNA helicase Hpr5p |

*^d^* Gene expression difference ratio of Log2 in LPS treated samples compared to control.
